# Supplementary material for: Gene expression and metabolic changes of Momordica charantia L. seedlings in response to low temperature stress
Source: PLoS One. 2020 May 29;15(5):e0233130. doi: 10.1371/journal.pone.0233130 (PMC7259686; doi:10.1371/journal.pone.0233130)
Supplement: Attachment — Submitted filename: PONE-D-19-35964_reviewer-20200218.pdf [file pone.0233130.s003.pdf]

PLOS ONE

Gene expression and metabolic changes of Momordica Charantia L. seedlings in response to low temperature stress

--Manuscript Draft--

**Manuscript Number:**	PONE-D-19-35964
**Article Type:**	Research Article
**Full Title:**	Gene expression and metabolic changes of Momordica Charantia L. seedlings in response to low temperature stress
**Short Title:**	Gene expression and metabolic changes in response to low temperature stress
**Corresponding Author:**	Yu Niu<br>tropical crops genetic resources research institution<br>danzhou, CHINA
**Keywords:**	crop breeding
**Abstract:**	Low temperature is the primary abiotic factor limiting germination, growth and distribution of the plant. Many tropical crops have been expanded plant latitude to pursue more economic value. Some of them are able to augment their chilling tolerance when exposed to the cold environment. Yet, the molecular and physiological mechanisms underlying this adaptive process still remain unknown. The objective of this study was to investigate the cold stress response in bitter gourd at phenotypic, physiological and metabolic levels. Here, we analysed the response of the cold-susceptible (Y17) and cold-resistant (Y54) bitter gourd seedlings to low temperature stress. Plant tissues were collected at different time points (0 to 24 h) and assayed for change in malondialdehyde (MDA), hydrogen peroxide (H_2O_2), proline content and activities of antioxidant enzymes. We also analysed the different expression of genes and metabolites in response to cold stress. Low temperature stress increased the accumulation of MDA, H_2O_2 and proline content in the bitter gourd. Exposing to the low temperature for 24 h caused the death of Y17 seedlings. Meanwhile, significant enhancing in antioxidant enzyme activities of POD (peroxidase), CAT (cataalase), SOD (superoxide dismutase) were observed. Differentially expressed genes analysis showed that genes encoded antioxidant enzymes and transcription factors increased their expression levels after exposing to the low temperature. The metabolite profiling demonstrated that many amino acid, sugar, fatty acid and organic acid metabolism play important roles in cold acclimation. These results suggested that the bitter gourd exhibited higher cold tolerance may attribute to the mobilization of antioxidant systems, the adjustment of the transcription factors and accumulation of osmoregulation substance. Compared with the cold-susceptible bitter gourd, the cold-resistant bitter gourd has a better performance in these aspects.
**Order of Authors:**	Yu Niu
	zi ji Liu
	huang He
	Xu Han
	zhi qiang Qi
	yan Yang
**Additional Information:**	
**Question**	**Response**
**Financial Disclosure**<br><br>Enter a financial disclosure statement that describes the sources of funding for the work included in this submission. Review the submission guidelines for detailed	he author(s) received no specific funding for this work.

Powered by Editorial Manager® and ProduXion Manager® from Aries Systems Corporation

requirements. View published research articles from *PLOS ONE* for specific examples.<br><br>This statement is required for submission and **will appear in the published article** if the submission is accepted. Please make sure it is accurate.<br><br>**Unfunded studies**<br>Enter: *The author(s) received no specific funding for this work.*<br><br>**Funded studies**<br>Enter a statement with the following details:<br>• Initials of the authors who received each award<br>• Grant numbers awarded to each author<br>• The full name of each funder<br>• URL of each funder website<br>• Did the sponsors or funders play any role in the study design, data collection and analysis, decision to publish, or preparation of the manuscript?<br>• **NO** - Include this sentence at the end of your statement: *The funders had no role in study design, data collection and analysis, decision to publish, or preparation of the manuscript.*<br>• **YES** - Specify the role(s) played.<br><br>* typeset	
**Competing Interests**<br><br>Use the instructions below to enter a competing interest statement for this submission. On behalf of all authors, disclose any competing interests that could be perceived to bias this work—acknowledging all financial support and any other relevant financial or non-financial competing interests.<br><br>This statement **will appear in the published article** if the submission is accepted. Please make sure it is accurate. View published research articles from *PLOS ONE* for specific examples.	The authors have declared that no competing interests exist.

Powered by Editorial Manager® and ProduXion Manager® from Aries Systems Corporation

**NO authors have competing interests**<br><br>Enter: *The authors have declared that no competing interests exist.*<br><br>**Authors with competing interests**<br><br>Enter competing interest details beginning with this statement:<br><br>*I have read the journal's policy and the authors of this manuscript have the following competing interests: [insert competing interests here]*<br><br>* typeset	
**Ethics Statement**<br><br>Enter an ethics statement for this submission. This statement is required if the study involved:<br><br>• Human participants<br>• Human specimens or tissue<br>• Vertebrate animals or cephalopods<br>• Vertebrate embryos or tissues<br>• Field research<br><br>Write "N/A" if the submission does not require an ethics statement.<br><br>General guidance is provided below. Consult the submission guidelines for detailed instructions. **Make sure that all information entered here is included in the Methods section of the manuscript.**	N/A

Powered by Editorial Manager® and ProduXion Manager® from Aries Systems Corporation

**Format for specific study types**<br><br>**Human Subject Research (involving human participants and/or tissue)**<br>• Give the name of the institutional review board or ethics committee that approved the study<br>• Include the approval number and/or a statement indicating approval of this research<br>• Indicate the form of consent obtained (written/oral) or the reason that consent was not obtained (e.g. the data were analyzed anonymously)<br><br>**Animal Research (involving vertebrate animals, embryos or tissues)**<br>• Provide the name of the Institutional Animal Care and Use Committee (IACUC) or other relevant ethics board that reviewed the study protocol, and indicate whether they approved this research or granted a formal waiver of ethical approval<br>• Include an approval number if one was obtained<br>• If the study involved *non-human primates*, add *additional details* about animal welfare and steps taken to ameliorate suffering<br>• If anesthesia, euthanasia, or any kind of animal sacrifice is part of the study, include briefly which substances and/or methods were applied<br><br>**Field Research**<br><br>Include the following details if this study involves the collection of plant, animal, or other materials from a natural setting:<br>• Field permit number<br>• Name of the institution or relevant body that granted permission	
**Data Availability**<br><br>Authors are required to make all data underlying the findings described fully available, without restriction, and from the time of publication. PLOS allows rare exceptions to address legal and ethical concerns. See the PLOS Data Policy and FAQ for detailed information.	Yes - all data are fully available without restriction

Powered by Editorial Manager® and ProduXion Manager® from Aries Systems Corporation

A Data Availability Statement describing where the data can be found is required at submission. Your answers to this question constitute the Data Availability Statement and **will be published in the article**, if accepted.<br><br>**Important:** Stating 'data available on request from the author' is not sufficient. If your data are only available upon request, select 'No' for the first question and explain your exceptional situation in the text box.<br><br>Do the authors confirm that all data underlying the findings described in their manuscript are fully available without restriction?	
**Describe where the data may be found in full sentences. If you are copying our sample text, replace any instances of XXX with the appropriate details.**<br><br>• If the data are **held or will be held in a public repository**, include URLs, accession numbers or DOIs. If this information will only be available after acceptance, indicate this by ticking the box below. For example: *All XXX files are available from the XXX database (accession number(s) XXX, XXX.)*.<br>• If the data are all contained **within the manuscript and/or Supporting Information files**, enter the following: *All relevant data are within the manuscript and its Supporting Information files.*<br>• If neither of these applies but you are able to provide **details of access elsewhere**, with or without limitations, please do so. For example:<br><br>*Data cannot be shared publicly because of [XXX]. Data are available from the XXX Institutional Data Access / Ethics Committee (contact via XXX) for researchers who meet the criteria for access to confidential data.*<br><br>*The data underlying the results presented in the study are available from (include the name of the third party*	All relevant data are within the manuscript and its Supporting Information files.

and contact information or URL).<br>• This text is appropriate if the data are owned by a third party and authors do not have permission to share the data.<br><br>* typeset	
Additional data availability information:	

Powered by Editorial Manager® and ProduXion Manager® from Aries Systems Corporation

Manuscript Click here to access/download;Manuscript;Manuscript.docx

Gene expression and metabolic changes of *Momordica Charantia* L. seedlings in response to low temperature stress

Yu Niu[1], Ziji Liu[1], Huang He[1], Xu Han[1], Zhiqiang Qi[1*], Yan Yang[1*]

1 Tropical Crops Genetic Resources Research Institute, CATAS, Danzhou Hainan 571737, China

*** Corresponding Author**

Yan Yang was the first and main corresponding author; yziqi@126.com

Zhiqiang Qi was the second corresponding author; zhiqiangqi@126.com

First Author

Yu Niu; niuyulong108@163.com

Ziji Liu; liuziji1982@163.com

Abstract: Low temperature is the primary abiotic factor limiting germination, growth and distribution of the plant. Many tropical crops have been expanded plant latitude to pursue more economic value. Some of them are able to augment their chilling tolerance when exposed to the cold environment. Yet, the molecular and physiological mechanisms underlying this adaptive process still remain unknown. The objective of this study was to investigate the cold stress response in bitter gourd at phenotypic, physiological and metabolic levels. Here, we analysed the response of the cold-susceptible (Y17) and cold-resistant (Y54) bitter gourd seedlings to low temperature stress. Plant tissues were collected at different time points (0 to 24 h) and assayed for change in malondialdehyde (MDA), hydrogen peroxide (H_2O_2), proline content and activities of antioxidant enzymes. We also analysed the different expression of genes and metabolites in response to cold stress. Low temperature stress increased the accumulation of MDA, H_2O_2 and proline content in the bitter gourd.

Exposing to the low temperature for 24 h caused the death of Y17 seedlings. Meanwhile, significant enhancing in antioxidant enzyme activities of POD (peroxidase), CAT (cataalase), SOD (superoxide dismutase) were observed. Differentially expressed genes analysis showed that genes encoded antioxidant enzymes and transcription factors increased their expression levels after exposing to the low temperature. The metabolite profiling demonstrated that many amino acid, sugar, fatty acid and organic acid metabolism play important roles in cold acclimation. These results suggested that the bitter gourd exhibited higher cold tolerance may attribute to the mobilization of antioxidant systems, the adjustment of the transcription factors and accumulation of osmoregulation substance. Compared with the cold-susceptible bitter gourd, the cold-resistant bitter gourd has a better performance in these aspects.

Introduction

Bitter gourd (*Momordica charantia* L.), a member of the Cucurbitaceae family, is a popular and widely grown vegetable in Southeast Asia due to its high level edible and medicinal value. It contains plenty of minerals, vitamin C and E, and is also used for modulating blood glucose and treating diabetes [1-2]. With increasing demand of bitter gourd in the Chinese market, plant regions of bitter gourd gradually extend towards the north. However, the low temperature of the spring and winter in most areas of the China can seriously restrict bitter gourd germination and growth, and then cause large decreases in the yield, because that bitter gourd is a tropical vegetable and chilling sensitive [3]. There is urgent need to breed some low-temperature tolerant cultivars to enlarge plant areas and guarantee market supply. Therefore, increasing cold resistance research of bitter gourd is very important.

Low temperature is the primary abiotic factor limiting germination, growth and distribution of the plant [4-5]. When subjecting to low temperature stress, plants usually evolve various physiological and biochemical changes and modulation of related gene expression to achieve cold acclimation, such as increasing quantities of antioxidant enzymes and antioxidants, osmotic solutes [6-7]. Low temperature stress

induces large accumulation of free radical, destroys the dynamic equilibrium of activate oxygen metabolism inside plant bodies, and poison cells [8-9]. Plants have increased enzymatic and non-enzymatic components to maintain active oxygen scavenging system, and reduced the toxicity of oxygen free radical to the cell, and decreased the destruction in cell structure, and improved cold tolerance of the plant [10]. One of the important changes caused by low temperature in plants is accumulation of osmoregulation substance. These substances can increase intracellular solute concentration, and lower ice point of the cell, and then reduce the injury of the low temperature to the cell [11-12]. However, there is little research regarding that how bitter gourd can adapt to the low temperature stress. Therefore, further research is still needed to clarify mechanism of resistance to low temperature for the bitter gourd.

Recently, transcriptome and metabonomics analysis have been extensively used in studying gene expression and metabolite changes of the crop to explore mechanism of cold resistance, such as rice, wheat and tomato [6, 13-14]. The research of Zhao et al. (2019) showed that fatty acid desaturases (FADs) play important roles in regulating fatty acid composition and maintaining membrane fluidity under temperature stress. The expression of genes encoding FADs (ZmFAD2.1&2.2, ZmFAD7, and ZmSLD1&3) in maize were significantly up-regulated under cold stress. According to the study that tomato plants exposed to 10 °C for 48 h， Barrero-Gil et al. (2016) found that tomato plants increased their tolerance to low temperature in a chilling acclimation process entailing comprehensive transcriptional and metabolic adjustments, including the accumulation of compatible solutes, the mobilization of antioxidant systems. However, few studies have been conducted from the transcription and metabolism level to explore cold acclimation mechanism of the bitter gourd.

In this study, cold-susceptible bitter gourd (Y17) and cold-resistant bitter gourd (Y54) were chosen and cultivated at low temperature of 5 °C for 24 h. The objective of this study was to reveal the cold acclimation mechanism of bitter gourd. A multidisciplinary approach, including phenotypic, physiological, genetic and

metabolic analysis, was used to reflect the response to cold stress in bitter gourd seedlings. These data could provide helpful information on understanding physiological mechanisms of bitter gourd underlying this adaptive response and providing a theoretical and practical basis for breeding low-temperature tolerant cultivars.

Materials and methods

Plant materials and growth conditions

Two bitter gourd cultivars (cold-susceptible (Y17) and cold-resistant (Y54)) were collected as the study materials. After germination, bitter gourd seedlings were grown in pots filled with sterilized soil and kept in a plant growth chamber under 12 h photoperiod with 60±5 % relative humidity, temperature 25±1 % and 150 µmol photons m^{-2} s^{-1} light intensity. When the seedlings grew to three leaves and one heart period, uniform bitter gourd seedlings (the number of each cultivar was 70) were placed in a growth chambers and subjected to low temperature treatment (5±1 °C) with light intensity of 150 µmol m^{-2} s^{-1} and 12 h photoperiod for 24 h. The leaves of six random samples of each bitter gourd cultivar were harvested after 0, 1, 2, 4, 8, 12 and 24 h of low temperature treatment for various assays. After harvest, the leaf samples were either used as fresh or were immediately frozen in liquid nitrogen and stored at -80 °C to further measurements.

Determination of physiological indices at different time interval

About 0.2 g fresh leaf segments collected from each pot at different time intervals were used for the determination of proline, superoxide dismutase (SOD), (peroxidase) POD, catalase (CAT), hydrogen peroxide (H_2O_2) and malondialdehyde (MDA) indicators. The proline content was determined using a sulfosalicylic acid assay. CAT activity was determined according to method of Wani et al. (2018). Concentration of MDA was assessed by a thiobarbituric acid colorimetric method [5]. POD activity was determine[illegible]e method as described by Zhang et al. (2005), i.e., the guaiacol method. SOD activity was estimated by the method of nitroblue tetrazolium assay [15]. H_2O_2 content was assayed as described by Si et al. (2017).

Gene expression analysis

For RNA extraction and cDNA synthesis, Total RNA was extracted from fresh leaves by using Trizol reagent (Invitrogen, Carlsbad, CA, USA) according to manufacturer's protocols, and then used DNase I (Roche) to purify total RNA. After extraction, RNA samples were utilized to construct the cDNA libraries, each library being generated from an equivalent mixture of three independent RNA preparations. Constructing the libraries and subsequent sequencing was performed by Illumina HiSeq TM 2000 technology in the company of the Beijing NovoGene.

Quantification of gene expression level: HTSeq v0.6.1 was used to count the reads numbers mapped to each gene. And then FPKM of each gene was calculated based on the length of the gene and reads count mapped to this gene. FPKM, expected number of Fragments Per Kilobase of transcript sequence per Millions base pairs sequenced, considers the effect of sequencing depth and gene length for the reads count at the same time, and is currently the most commonly used method for estimating gene expression levels [17].

Differentially expressed genes: Differential expression analysis of two groups was performed using the DESeq R package (1.18.0). DESeq provide statistical routines for determining differential expression in digital gene expression data using a model based on the negative binomial distribution. The resulting P-values were adjusted using the Benjamini and Hochberg's approach for controlling the false discovery rate. Genes with an adjusted *p*-value <0.05 found by DESeq were assigned as differentially expressed.

Metabolite extraction and metabolite profiling analysis

For each bitter gourd variety, the leaves of six random samples at 0 h, 8 h and 24 h of the treatment were chosen to analyse metabolite changes. After freeze-drying, the leaf tissue was grinded to powders. Approximately 150 mg tissue powders were transferred into 2 ml centrifuge tubes. 1mL mixed solution contained methanol, acetonitrile and water (2:2:1, v/v/v) was added to the tubes. The mixtures were vortexed at low temperature for 30 min, twice. Subsequently, the tubes was placed at -20 °C for 1 h. The supernatant was decanted into a new glass tube and was freeze-dry

after the tubes were centrifuged at 13,000 rpm at 4 °C for 15 min. Then, the samples were saved at a -80 °C place. Before GC-MS analysis, 100 uL of mixed solution contained acetonitrile and water (1:1, v/v) to the samples to vortex. After the tubes were centrifuged at 14,000 rpm at 4 °C for 15 min, the supernatant was extracted to analysis. The samples were placed in the 4 °C autosampler during all analysis process. Agilent 1290 Infinity LC Systems was used to separate and chromatographic column HILIC was used to separate. The injection volume was 2 uL, the flow rate was 0.3 mL/min, and the column temperature was 25 °C. The mobile phase A was water-25 Mm ammonium acetate-25 Mm ammonia water. Mobile phase B was acetonitrile.

Original data was converted into the format of mzXML by ProteoWizard. Then XCMS program was used to extract peak area. Metabolites were identified by searching the database established by our laboratory. SIMCA-P 14.1 software package (Umetrics, Umea, Sweden) was conducted a principal component analysis (PCA), partial least squares discriminant analysis (PLS-DA), orthogonal partial least squares discriminant analysis (OPLS-DA) after the data treated by Pareto-scaling. PLS-DA and OPLS-DA analyses were used to obtain variable importance values (VIP). Differential metabolites were found using Student's *t* test (p<0.05) and VIP (VIP>1.2).

Statistical analysis

In this study, comparisons between two groups were made utilizing the Student's t-test. Spearman's correlation was performed to analyse the correlation by SPSS 18.0. A p value<0.05 was used to test for statistical significance.

Results

Morphology changes in response to low temperature stress

In this study, the low temperature had a remarkable influence on the growth of bitter gourd seedlings. As shown in Figure 1, the damage of low temperature to the cold-susceptible bitter gourd Y17 seedlings was obvious after 2 h of 5 °C exposure. The leaves of bitter gourd Y17 seedlings began to appear the phenomenon of roll and water loss. Many of the bitter gourd Y17 seedlings died when low temperature

treatment was prolonged for 24 h. However, the obvious damage of low temperature on the morphology of the cold-resistant bitter gourd Y54 seedlings appeared after exposing to [illegible] temperature for 8 h. After 24 h of the treatment, bitter gourd Y54 seedlings were still survival an[illegible]ut the leaves were roll and water[illegible]s.

Changes in MDA and H_2O_2 content in response to low temperature

With increasing times exposed to low temperature, MDA and H_2O_2 content in two bitter gourds had an increasing trend (Fig 2). After 24 h of low temperature treatment, MDA content of the bitter gourd Y17 and Y54 were increased 97.80% and 89.29%[illegible]compare with that before the treatment, respectively ($P < 0.05$). After 8 h, 12 h and 24 h of the treatment, the cold-resistant bitter gourd Y54 all had a lower MDA and H_2O_2 content than the cold-susceptible bitter gourd Y17 ($P < 0.05$). MDA content of the bitter gourd Y54 was 3.65±0.51 umol g^{-1} after 24 h for the low temperature treatment, which was lower than that of bitter gourd Y17 by 19.41%. Maximum contents of H_2O_2 were observed in the Y17 after 24 h cold treatment by 2.48±0.074 mmol g^{-1}, which was higher than that of bitter gourd Y54 by 8.13% ($P <$ 0.05).

Changes in enzymatic activities and proline content in response to low temperature

Figure 3 shows the POD, SOD and CAT activities and the proline content of the bitter gourd seedlings at different time points of low temperature treatment. With the passage of time exposed to low temperature, [illegible]ndexes of bitter gourd Y17 and Y54 were observe[illegible]n increasing trend, especially after 8 h of the treatment. After 24 h of the cold treatment, POD, SOD and CAT activity of the bitter gourd Y17 were 101.67±9.60 U $g^{-1}min^{-1}$, 219.48±2.52 U g^{-1} and 26.47±0.017 U g^{-1}, which were significantly higher than that before the treatment by 69.44%, 100.02% and 69.61%, respectively ($P < 0.05$).

Compared with the bitter gourd Y17, the bitter gourd Y54 had a significant higher POD, SOD and CAT activities and the proline content after exposing to low temperature. The POD activity of Y54 was found to be 61.67±2.45 U $g^{-1}min^{-1}$ at the beginning to exposed to low temperature, while it increased by 40.54% after 4 h,

72.97% after 8 h and 104.32% after 24 h of 5 °C exposure (Fig 3A). The POD activity of the bitter gourd Y54 was signific[illegible] higher than that of bitter gourd Y17 by 23.93% after 24 h of the low temperature treatment (*P* < 0.05). The SOD activity of Y54 at 5 °C was found to be 103.09±5.17 U g^{-1} at 0 h of the treatment, but this activity was increased to 225.64±2.31 U g^{-1} after plant grown at 5 °C for 24 h (Fig 3B). Compared with CAT activity at th[illegible]ginning treatment, CAT activity of Y54 at the time point of 24 h was elevated with a[illegible]creasing of 81.73%, and which was also higher than that of the bitter gourd Y17 by 9.25% (Fig 3C). After growing at 5 °C for 24 h, the proline content of Y54 was 51.16±1.58 mg g^{-1} and was increased by 114.32% to compare with that of 0 h and higher than that of bitter gourd Y17 by 17.85% (Fig 3D).

H[illegible] content of the bitter gourd showed a significant positive correlation with MDA content (r=0.48, *P* < 0.05), with POD activity (r=0.54, *P* < 0.05), with SOD activity (r=0.54, *P* < 0.05). But no significant correlation was found between H_2O_2 content and CAT activity (Tab 1).

Tab[illegible] the relationship of physiological indexes

	Proline	MDA	SOD	POD	CAT	H_2O_2
Proline	1	0	0	0	0	0
MDA	0.17	1	0	0	0	0
SOD	0.56*	0.042	1	0	0	0
POD	0.64**	0.28	0.80**	1	0	0
CAT	0.50*	-0.16	0.053	0.25	1	0
H_2O_2	0.53*	0.48*	0.54*	0.54*	-0.085	1

* Indicates a significant difference at the 0.05 level. ** Indicates a significant difference at the 0.01 level.

Differentially expressed genes in response to low temperature stress

In this study, [illegible]y 46 differentially expressed genes associated with cold acclimation were selected and annotated after the bitter gourd exposed at 5 °C (Figure 4). Among them, 69.57% (n=32) of genes encoded transcription factors, 8.70% (n=4) of genes encoded peroxidase, and others encoded SOD, sucrose synthase, pyruvate decarboxylase, glutamate dehydrogenase, chalcone synthase, phosphofructokinase, nerolidol synthase and germacrene D synthase. Compared with 0 h of the treatment, four POD genes in the Y54 showed 5.01-25.45 fold increased gene expression level at

8 h of low temperature treatment, and 6.97-452.58 fold increased gene expression level at 24 h of the treatment. However, three of them in the Y 17 showed increased gene expression level at 8 h of the treatment, and increased levels ranged from 0.28-1.05 fold. Continued low temperature for 24 h led to down-regulated expression of three POD genes. SOD gene of the bitter gourd Y17 showed 1.36 and 5.52 fold increased gene expression level, respectively, at 8 h and 24 h of low temperature treatment. In the bitter gourd Y54, gene expression level of SOD gene increased 2.35 and 11.02 fold. After 8 h of low temperature, there were 27 and 23 up-regulated expression of transcription factor genes in the bitter gourd Y54 and Y17, respectively. While the numbers of up-regulated expression genes changed to 27 and 25 after 24 h of the low temperature treatment. The elevated gene expression levels that were higher in the Y54 than that in the Y17 consisted of mainly transcription factor after exposing to low temperature for 8 h and 24 h, including McERF, McMYB, McWRKY, McTCP, McRAX, McPCL, McGATA, McE2F and McbZIP (Fig 4).

Differentially expressed metabolites in response to low temperature stress

Based on the metabolic responses of bitter gourd Y54 and Y17 under low temperature stress, 195 differential expressed metabolites, including 48 amino acids, 2 polypeptides, 35 sugars, 10 fatty acids, 38 organic acids, 11 flavonoids, 7 nucleobases, 4 coenzyme, 1 steride and 39 other compounds were screened and analyzed. Among them, 132 metabolites had greater accumulation levels in the bitter gourd Y54 and Y17 after 8 h and 24 h of the low temperature treatment, including 37 amino acids, 2 polypeptides, 33 sugars, 3 fatty acids, 22 organic acids, 1 flavonoids, 7 nucleobases, 3 coenzyme, 1 steride and 22 other compounds. Compared with the bitter gourd Y17, 115 metabolites, including 35 amino acids, 24 sugars, 10 fatty acid, 19 organic acids, 1 flavonoid, 7 nucleobase, 2 coenzyme, 1 steride and 16 other compounds, had greater accumulation levels in Y54 after exposing to low temperature for 8 h and 24 h. A further comparative analysis indicated the main difference expression of sugar in the bitter gourd Y54 and Y17 were sucrose and raffinose (Fig 5).

Discussion

Damages of low temperature stress on the growth of bitter gourd seedlings

Low temperature is a major environmental stress that seriously affects plant growth, distribution and productivity [4]. Many crops originate from tropical origin and are highly sensitive to low non-freezing temperatures due to their bad chilling adjustment ability [6]. In this work, our results showed that low temperature had a serious damage to the growth of bitter gourd. Sustained low temperature exposing even caused the (death of cold-susceptible bitter gourd seedlings (Figure 1). Plant cells produce excess of reactive oxygen species (ROS) under low temperature stress, and then lead to the accumulation of MDA. MDA is produced in membranes and can destroy membrane structure and cause protein degradation, and then aggravates the damage of low temperature [18-19]. In the low temperature treatment, MDA in the bitter gourd Y17 had an increasing trend with increasing time exposed to the low temperature. Content of MDA increased almost 1 fold after 24 h of low temperature stress (Fig 2). Compared with the performance of cold-susceptible bitter gourd, the cold-resistant bitter gourd Y54 had a good ability to to[illegible]nt low temperature stress (Fig 1). After 24 h of low temperature stress, the leaves of bitter gourd Y54 were only roll and water loss. We found that this adaptive response included extensive transcriptional and metabolic adjustments, such as the accumulation of osmoregulation substance, the mobilization of antioxidant systems and the increasing expression of transcription factor genes.

Cold acclimation in bitter gourd results in mobilization of antioxidant mechanisms

For keeping the balance of ROS numbers, plants have evolved some enzymatic and non-enzymatic antioxidant systems to eliminate or reduce the ROS damage induced by low temperature stress [4, 20]. SOD, POD and CAT are considered as the most important antioxidant enzymes to eliminate ROS. The main function of SOD is converting highly reactive O_2^- to O_2 and H_2O_2. POD, CAT can disintegrate produced H_2O_2 into water and molecular oxygen [21-22]. Plants usually elevate antioxidant enzyme levels to tolerance various environmental stresses [4, 9]. Similarly, the significant enhances of the activity of POD, SOD and CAT was detected in the bitter gourd Y17 and Y54 after exposure to low temperature for 24 h (Fig 3). The

phenomenon of elevated antioxidant enzyme activities also can be found other crops when they exposed to different environmental stresses, such as maize [23] and tomato [24]. This is a normal defensive response of the plant to low temperature stress, which is benefit to the plant with higher cold tolerance to survival in the colder environment and a long time of low temperature stress [25-26]. The correlation analysis also showed that there was a significant positive correlation between H_2O_2 content and MDA content, SOD and POD activities (Tab 1). In this study, low temperature stress causes the accumulation of MDA content, which leads to the bitter gourd elevates the SOD activity to convert ROS to H_2O_2, and then enhances the activity of POD and CAT to eliminate H_2O_2.

The antioxidant defense system involved in resisting low temperature stress in bitter gourd could also be detected at the transcript level (Fig 3). In this study, POD genes displayed an increased transcript expression in the bitter gourd Y54 after 8 h and 24 h of the low temperature treatment. Significant increase in POD gene expression was also reported during low temperature stress in case of bermudagrass [10]. The expression level of genes encoding SOD enzyme in the bitter gourd Y54 increased 2 to 11 fold after exposing to the low temperature. Similarly, low temperature stress is reported to up-regulate the expression of SOD in rice [27] and bermudagrass [28].

Compared with the cold-susceptible bitter gourd Y17, the MDA and H_2O_2 content of the cold-resistant bitter gourd Y54 were significantly lower after low temperature treatment (Fig 2). It implies that damages of cold stress to the bitter gourd Y54 is smaller than that of bitter gourd Y17. The result showed that the reason for the cold-resistant bitter gourd has better ability to adapt to low temperature stress is attributed to its higher antioxidant enzyme gene expression level and activities in response to low temperature (Fig 3, 4). Compared with that of the bitter gourd Y17, POD, SOD and CAT activities of the bitter gourd Y54 increased by 2.80% - 23.93% after 24 h of low temperature stress. The expression of three POD genes was down-regulated at the time point of 24 h of the treatment, while all POD genes were up-regulated expression. Therefore, better antioxidant defense system can help the

plant to preferably adapt to the low temperature stress.

Cold acclimation response in bitter gourd involves transcription factors associated with cold acclimation

Transcription factor is a critical element of all gene regulatory networks that mediate various aspects of plant growth and developmental processes. It also plays a central role in controlling the expression of cold-responsive genes with a role in cold acclimation [29-30]. The result showed that the expression levels of most transcription factor genes were up-regulated in the bitter gourd subjected to 5 °C for 8 h and 24 h (Fig 4). These findings strongly suggested that the transcription factor plays an important role in cold acclimation process in bitter gourd. These differentially expressed genes associated with cold acclimation encoded transcription factors also can find in other cold tolerant plant [6, 31]. The research of Satoh et al. (2004) found that bZIPs transcription factor have potential ability to activate expression of ProDH gene, which participate in the metabolism of proline in Arabidopsis plants. In this study, the expression levels of McbZIP1 and McbZIP2 transcription factor of the bitter gourd Y54 were up-regulated after exposing to the low temperature (Fig 4). It suggests that McbZIPs transcription factor participates in the response of the bitter gourd to low temperature stress. WRKY transcription factor genes have been known that playing important roles in the regulation of transcriptional reprogramming associated with plant stress responses [31]. For example, transgenic Arabidopsis plants overexpressing GmWRKY21 showed increased tolerance to cold stress when compared with wild-type plants [33]. This study found that four WRKY transcription factor genes all elevated the transcription expression level after exposing to the low temperature. Therefore, it indicates that the WRKY transcription factors play an important role in promoting the bitter gourd to resist low temperature stress. Some research have demonstrated that the family of MYB transcription factor involves in the expression of ABA-dependent gene and CBF genes, and then influence the performance of the plant to resist low temperature stress [31, 34]. Six MYB transcription factor gene up-regulated expression levels after 8 h and 24 h of the low temperature treatment, which means that these transcript

factor have a close tine to the cold tolerance mechanism in the bitter gourd.

Cold-induced osmoregulation substance expression

In response to the low temperature stress, the plant accumulated a range of osmoregulation substance to resist the cold stress, including sugar, fatty acid and amino acid [11, 31]. A lot of evidence has demonstrated that there is a significant positive correlation between the accumulation of proline and improved cold tolerance in the plant [6, 35]. Proline can regulate cytosol acidity and decrease lipid peroxidation to improve plant tolerance of low temperature stress [36]. In this study, there was an obvious increasing trend of the proline content in the bitter gourd after exposing to the low temperature stress (Fig 3). It suggests that the bitter has the ability to improve the proline content to resist the cold stress. We also found that the glutamate which participated in synthesizing the proline was significant expression after exposing to the low temperature stress (Fig 5). In addition, the cold-resistant bitter gourd Y54 could accumulate more proline than the cold-susceptible bitter gourd Y17 after 2 h of the treatment, which implies that the cold-resistant bitter gourd has a faster and better chilling acclimation to cold stress.

Carbohydrate metabolism have been reported to be a main contributed process of chilling acclimation, and the precursor and intermediate products of carbohydrate compounds all can play roles as osmoregulators, cryoprotectants or signaling molecules [37-38]. Soluble carbohydrate can regulate cell osmotic potential to maintain the balance of osmotic pressure, and protect plasma membrane during cold stress [39]. In this study, 33 sugars had up-regulated expression levels in the bitter gourd after 8 h and 24 h of the low temperature treatment. These up-regulations suggest that there is a tight link between carbohydrate metabolism and cold stress. The bitter gourd can adjustment carbohydrate metabolism to tolerant cold stress. The accumulation of sucrose as an osmoprotectant supports the role of the plant to stabilize cellular membranes and maintains turgor, when they exposed to low temperature stress [40]. The metabolite profiling analysis showed that the sucrose had greater accumulation levels in the bitter gourd at 8 h and 24 h of the low temperature treatment, meanwhile the expression level of sucrose synthase was up-regulated,

which suggests that the sucrose metabolism also has an important role in chilling acclimation of the biter gourd. After exposing to the low temperature stress for 8 h and 24 h, the expression level of raffinose in the bitter gourd has significantly elevated (Fig 5). This phenomenon of increased raffinose content also can be found in other plant subjected to cold stress, such as maize, Siberian spruce [41-42]. The role of the raffinose has been implicated in membrane protection and radical scavenging, and the accumulation of raffinose content is contributed to elevate cold tolerance of the plant [43-44]. Therefore, carbohydrate metabolism plays an important role in chilling acclimation of the bitter gourd.

Conclusions

In this study, the result demonstrated that the low temperature stress had obvious damages to the growth of the chilling sensitive bitter gourd. However, the bitter gourd also developed some protected mechanisms to tolerance the cold stress. The defense mechanisms included the accumulation of osmoregulation substance, such as amino acid, sugar, fatty acid and organic acid, and the mobilization of antioxidant systems, such as up-regulating expression levels of genes related cold tolerant and increasing antioxidant enzyme activities. In addition, chilling acclimation was regulated by a similar set of transcription factors, including bZIP, MYB and WRKY family. The cold-resistant bitter gourd has a better performance than the cold-susceptible bitter gourd in these aspects. Based on the result, we expect that this work can provide new knowledge and idea to breeding cold tolerance bitter gourd.

Acknowledgments

This work was financially supported by Key Research and Development Projects of Hainan Province（ZDYF2019103）and National Nonprofit Institute Research Grant of CATAS-TCGRI (1630032017027).

Author Contributions

Writing - original draft: Yu Niu, Ziji Liu, Yan Yang.

Writing – review & editing: Yan Yang, Zhiqiang Qi.

Performed the experiments: Huang He.

Analyzed the data: Xu Han

References

1. Lucas E A, Dumancas G G, Smith B J, Clarke S L, Arjmandi B H. Chapter 35 -Health Benefits of Bitter Melon (Momordica charantia). In R. R. W. R. Preedy (Ed.), Bioactive Foods in Promoting Health. San Diego: Academic Press. 2010. pp. 525-549.
2. Saini R, Rai K P, Bara M B, Sahu P, Anjer T, Kumar R. Effect of different seed priming treatments and its duration on seedling characters of Bitter gourd (*Momordica charantia* L.). Journal of Pharmacognosy and Phytochemistry. 2017; 6(5): 848-850.
3. Wang J N, Shang S, Tian L B, Zhou M M, Pan Q Y, Zou K X, Du Z T. Effects of low temperature stress on antioxidant system of grafted bitter gourd seedlings. Chinese Journal of Tropical Crops. 2018; 39(2): 237-245. (With Chinese abstract)
4. Wani W A, Jan N, Qazi H A, Andrabi K I, John R. Cold stress induces biochemical changes, fatty acid profile, antioxidant system and gene expression in Capsella bursa pastoris L.. Acta Physiologiae Plantarum. 2018; 40: 167.
5. Peng M W, Chang Y L, Chu G M, Wang M. Low-temperature tolerance and transcriptome analyses during seed germination of Anabasis aphylla. Journal of Plant Interactions. 2019; 14(1): 254-264.
6. Barrero-Gil J, Huertas R, Rambla LJ, Granell A, Salinas J. Tomato plants increase their tolerance to low temperature in a chilling acclimation process entailing comprehensive transcriptional and metabolic adjustments. Plant, Cell and Environment. 2016; 39: 2303-2318.
7. Li S M, Yang Y, Zhang Q, Liu N F, Xu Q G, Hu L X. Differential physiological and metabolic response to low temperature in two zoysiagrass genotypes native to high and low latitude. PLoS ONE. 2018; 13(6): e0198885. https://doi.org/10.1371/journal.pone.0198885
8. Caverzan A, Passaia G, Rosa S B, Ribeiro C W, Lazzarotto F, Margis-Pinheiro M.

Plant responses to stresses: role of ascorbate peroxidase in the antioxidant protection. Genetics and Molecular Biology. 2012; 35(4): 1011-1019.

9. Raja V, Majeed U, Kang H, Andrabi KI, John R. Abiotic stress: interplay between ROS, hormones and MAPKs. Environmental and Experimental Botany. 2017; 137: 142-157.
10. Liu A, Hu Z G, Bi A Y, Fan J B, Gitau M M, Amombo E, et al. Photosynthesis, antioxidant system and gene expression of bermudagrass in response to low temperature and salt stress. Ecotoxicology. 2016; 25(8): 1445-1457.
11. Ruelland E, Vaultier M N, Zachowski A, Hurry V, Kader J C, Delseny M. Cold signalling and cold acclimation in plants. Advances in Botanical Research. 2009; 49: 35-150.
12. Theocharis A, Clément C, Barka E A. Physiological and molecular changes in plants grown at low temperatures. Planta. 2012; 235: 1091-1105.
13. Zhao J L, Zhang S H, Yang T F, Zeng Z C, Huang Z H, Liu Q, et al. Global transcriptional profiling of a cold-tolerant rice variety under moderate cold stress reveals different cold stress response mechanisms. Physiologia Plantarum. 2015; 154(3): 381-394.
14. Zhao X C, Wei J P, He L, Zhang Y F, Zhao Y, Xu X X, et al. Identification of fatty acid desaturases in maize and their differential responses to low and high temperature. Genes. 2019; 10: 445.
15. Zhang H, Jiang Y, He Z, Ma M. Cadmium accumulation and oxidative burst in garlic (Allium sativum). Journal of Plant Physiology. 2005; 162: 977-984.
16. Si T, Wang X, Wu L, Zhao C Z, Zhang L N, Huang M, et al. Nitric Oxide and Hydrogen Peroxide Mediate Wounding-Induced Freezing Tolerance through Modifications in Photosystem and Antioxidant System in Wheat. Frontiers in Plant Science. 2017; 8: 1284.
17. Trapnell C, Williams B A, Pertea G, Mortazavi A, Kwan G, van Baren M J, et al. Transcript assembly and quantification by RNA-Seq reveals unannotated transcripts and isoform switching during cell differentiation. Nature Biotechnology. 2010; 28: 511-515.

18. Langridge P, Paltridge N, Fincher G. Functional genomics of abiotic stress tolerance in cereals. Briefings in Functional Genomics & Proteomics. 2006; 4: 343-354.

19. Martinez V, Nieves-Cordones M, Lopez-Delacalle M, Rodenas R, Mestre T C, Garcia-Sanchez F, et al. Tolerance to stress combination in tomato plants: new insights in the protective role of melatonin. Molecules. 2018; 23(3): 535.

20. Petrov V, Hille J, Mueller-Roeber B, Gechev T S. ROS-mediated abiotic stress-induced programmed cell death in plants. Frontiers in Plant Science. 2015; 6: 69.

21. Wang F, Liu J, Zhou L, Pan G, Li Z, Cheng F. Senescence-specific change in ROS scavenging enzyme activities and regulation of various SOD isozymes to ROS levels in psf mutant rice leaves. Plant Physiology and Biochemisrty. 2016; 109: 248-261.

22. Jan N, Majeed U, Andrabi K I, John R. Cold stress modulates osmolytes and antioxidant system in Calendula officinalis. Acta Physiologiae Plantarum. 2018, 40: 73.

23. Zhu X C, Song F B, Xu H W. Influence of arbuscular mycorrhiza on lipid peroxidation and antioxidant enzyme activity of maize plants under temperature stress. Mycorrhiza. 2010; 20: 325-332.

24. Ogweno J O, Song X S, Hu W H, Shi K, Zhou Y H, Yu J Q. Detached leaves of tomato differ in their photosynthetic physiological response to moderate high and low temperature stress. Scientia Horticulturae. 2009; 123(1): 17-22.

25. Baek K H, Skinner D Z. Alteration of antioxidant enzyme gene expression during cold acclimation of near-isogenic wheat lines. Plant Science. 2003; 165(6): 1221-1227.

26. Kuk Y I, Shin J S, Burgos N R, Hwang T E, Han O, Cho B H, et al. Antioxidative enzymes offer protection from chilling damage in rice plants. Crop Science. 2003; 43(6): 2109-2117.

27. Nanculao G D, Herrera M L, Carcamo M P, Velasquez V B. Relative expression of genes related with cold tolerance in temperate rice at the seedling stage. African

Journal of Biotechnology. 2014; 13(25): 2506-2512.

28. Fan J, Ren J, Zhu W, Amombo E, Fu J, Chen L. Antioxidant responses and gene expression in bermudagrass under cold stress. American Society for Horticultural Science. 2014; 139(6): 699-705.

29. Van Buskirk H A, Thomashow M F. Arabidopsis transcription factors regulating cold acclimation. Physiologia Plantarum. 2006; 126: 72-80.

30. Seo P J, Park M J, Park C M. Alternative splicing of transcription factors in plant responses to low temperature stress: mechanisms and functions. Planta. 2013; 237: 1415-1424.

31. Chen L G, Song Y, Li S J, Zhang L P, Zou C S, Yu D Q. The role of WRKY transcription factors in plant abiotic stresses. Biochimica et Biophysica Acta. 2012; 1819(2): 120-128.

32. Satoh R, Fujita Y, Nakashima K, Shinozaki K, Yamaguchi-Shinozaki K. A novel subgroup of bZIP proteins functions as transcriptional activators in hypoosmolarity-responsive expression of the *ProDH* gene in *Arabidopsis*. Plant & Cell Physiology. 2004; 45: 309-317.

33. Zhou Q Y, Tian A G, Zou H F, Xie Z M, Lei G, Huang J, et al. Soybean WRKY-type transcription factor genes, *GmWRKY13*, *GmWRKY21*, and *GmWRKY54*, confer differential tolerance to abiotic stresses in transgenic Arabidopsis plants. Plant Biotechnology Journal. 2008; 6: 486-503.

34. Agarwal M, Hao Y J, Kapoor A, Dong C H, Fujii H, Zheng X W, et al. A R2R3 type MYB transcription factor is involved in the cold regulation of CBF genes and in acquired freezing tolerance. The Journal of Biological Chemistry. 2006; 281: 37636-37645.

35. Szabados L, Savouré A. Proline: a multifunctional amino acid. Trends in Plant Science. 2010; 15: 89-97.

36. Kavi Kishor P B, Sangam S, Amrutha R N, Sri Laxmi P, Naidu K R, Rao K R S S, et al. Regulation of proline biosynthesis, degradation, uptake and transport in higher plants: Its implications in plant growth and abiotic stress tolerance. Current Science. 2005; 88: 424-438.

37. Welling A, Palva E T. Molecular control of cold acclimation in trees. Physiologia Plantarum. 2006, 127: 167-181.

38. Fernandez O, Theocharis A, Bordiec S, Feil R, Jacquens L, Clément C, Fontaine F, Ait Barka E. Burkholderia phytofirmans strain PsJN acclimates grapevine to cold by modulating carbohydrates metabolism. International Society of Molecular Plant-Microbe Interactions. 2012; 25(4): 496-504.

39. Bogdanović J, Mojović M, Milosavić N, Mitrović A, Vučinić Ž, Spasojević I. Role of fructose in the adaptation of plants to cold-induced oxidative stress. European Biophysics Jouranl. 2008; 37(7): 1241-1246.

40. Mundree S.G., Baker B., Mowla S., Peters S., Marais S., Wilingen C.V., et al. Physiological and molecular insights into drought tolerance. African Journal of Biotechnology. 2002; 1(2): 28-38.

41. Angelcheva L, Mishra Y, Antti H, Kjellsen T D, Funk C, Strimbeck R G, et al. Metabolomic analysis of extreme freezing tolerance in Siberian spruce (Picea obovata). New Phytologist. 2014; 204(3): 545-555.

42. Shahryar N, Maaliamiri R. Metabolic acclimation of tetraploid and hexaploid wheats by cold stress-induced carbohydrate accumulation. Journal of Plant Physiology. 2016; 204: 44-53.

43. Hincha D K. Effects of calcium-induced aggregation on the physical stability of liposomes containing plant glycolipids. Biochimica et Biophysica Acta - Biomembranes. 2003; 1611(1-2): 180-186.

44. Nishizawa A, Yabuta Y, Shigeoka S. Galactinol and raffinose constitute a novel function to protect plants from oxidative damage. Plant Physiology. 2008; 147: 1251-1263.

Figure

Figure 1 Performance at the seedling stage of cold-susceptible bitter gourd Y17 and cold-resistant bitter gourd Y54 after low temperature treatment

Figure 2 Effect of cold stress on MDA and H_2O_2 content in bitter gourd seedlings at different time points. The data represent means ± SE. Bars with different letters are significantly different at $P < 0.05$ in the same time point of the cold treatment.

Figure 3 Effect of cold stress on POD, SOD and CAT activities and proline content in bitter gourd seedlings at different time points. The data represent means ± SE. Bars with different letters are significantly different at $P < 0.05$ in the same time point of the cold treatment.

Figure 4 Effects of low temperature on the cold induced gene expressions in leaves of bitter gourd

Figure 5 Differentially expressed metabolites of bitter gourd in response to low temperature stress

New Manuscript
Click here to access/download;Manuscript;New Manuscript.docx

Gene expression and metabolic changes of *Momordica Charantia* L. seedlings in response to low temperature stress

Yu Niu[1,#], Ziji Liu[1,#], Huang He[1], Xu Han[1], Zhiqiang Qi[1,*], Yan Yang[1,*]

1 Tropical Crops Genetic Resources Research Institute, CATAS, Danzhou Hainan 571737, China

[*] *Corresponding to: Prof. Yan Yang, Tropical Crops Genetic Resources Research Institute, CATAS, Danzhou Hainan 571737, China. Tel: +86 0898-23306575, fax: +86 0898-23306210 E-mail: yziqi@126.com; Prof. Zhiqiang Qi, Tropical Crops Genetic Resources Research Institute, CATAS, Danzhou Hainan 571737, China. Tel: +86 0898-23306575, fax: +86 0898-23306210 E-mail: zhiqiangqi@126.com.*

[#] The author contributed equally to this work

Abstract: Low temperature is one of the abiotic factors limiting germination, growth and distribution of the plant in current plant-products industry, especially for the tropical vegetables in non-tropical area or tropical fields under cold temperature. Screening the plant with ability of anti-cold temperature captured worldwide attention and exerted great importance. In our previous work, the anti-cold species of *Momordica Charantia* L. seedlings was screened out. Yet, the molecular and physiological mechanisms underlying this adaptive process still remain unknown. This study was aimed to investigate the mechanism of anti-cold species of *Momordica Charantia* L. in genetical and metabolomics levels. Metabolomics and transcriptomics method were applied to characterized the changes of initial metabolites and genes, respectively. Two species, including cold-susceptible group (Y17) and cold-resistant group (Y54) were evaluated, including indexes of malondialdehyde (MDA), hydrogen peroxide (H_2O_2), proline content, activities of antioxidant enzymes, metabolites changes and genes differentiation in plant tissues at different time points (0 to 24 h). It found that low temperature stress resulted in increased accumulation of MDA, H_2O_2 and proline content in two species bitter gourd, but these expressions in Y54 was less than Y17. As compared to Y17, Y54 presented significantly enhanced antioxidant enzyme activities of POD (peroxidase), CAT (cataalase) and SOD (superoxide dismutase). Genes encoded antioxidant enzymes and transcription factors were dramatically increased after exposing to the low temperature and Y54 exerted the higher expression than Y17. The metabolomic results demonstrated that amino acid, sugar, fatty acid and organic acid metabolism played important roles in cold acclimation. These results suggested that the bitter gourd with anti-cold ability might attribute to mobilization of antioxidant systems, adjustment of the transcription factors and accumulation of osmoregulation substance to achieve higher cold tolerance. This work presented meaning information for revealing the anti-cold mechanism of the bitter gourd and new insight for further screening of anti-cold species in other plant.

Introduction

Low temperature is the primary abiotic factor limiting germination, growth and distribution of the plant [1-2]. When subjecting to low temperature stress, plants usually evolve various physiological and biochemical changes and modulation of related gene expression to achieve cold acclimation, such as increasing quantities of antioxidant enzymes and antioxidants, osmotic solutes [3-4]. Low temperature stress induces large accumulation of free radical, destroys the dynamic equilibrium of activate oxygen metabolism inside plant bodies, and poison cells [5-6]. Plants have increased enzymatic and non-enzymatic components to maintain active oxygen scavenging system, and reduced the toxicity of oxygen free radical to the cell, and decreased the destruction in cell structure, and improved cold tolerance of the plant [7]. One of the important changes caused by low temperature in plants is accumulation of osmoregulation substance. These substances can increase intracellular solute concentration, and lower ice point of the cell, and then reduce the injury of the low temperature to the cell [8-9]. However, there is little research regarding that how bitter gourd can adapt to the low temperature stress. Therefore, further research is still needed to clarify mechanism of resistance to low temperature for the bitter gourd.

Bitter gourd (*Momordica charantia* L.), a member of the Cucurbitaceae family, is a popular and widely grown vegetable in Southeast Asia due to its high-level edible and medicinal values. It contains plenty of minerals, vitamin C and E, *etc* [10]. It was also applied for modulating blood glucose and treating diabetes in our daily life [11]. With increasing demand of bitter gourd in the Chinese market, plant regions of bitter gourd gradually were extended from south to north in order to meet the customers' requirement. However, the low temperature in spring and winter or most areas of the China seriously restricted germination and growth of bitter gourd since it is a tropical vegetable and chilling sensitive [12]. Thus, it was urgent need to breed some low-temperature tolerant cultivars to enlarge plant areas and guarantee market supplies. In our previous work, the anti-cold species of bitter gourd was found [13]. However, the mechanism of their anti-cold ability was still unsolved.

Recently, transcriptome and metabolomics analysis had been extensively used in

fields of crops to explore mechanism of cold resistance, such as rice, wheat and tomato [6, 14-15]. Zhao et al. (2019) found that fatty acid desaturases (FADs) play important roles in regulating fatty acid composition and maintaining membrane fluidity under temperature stress and the expression of genes encoding FADs (ZmFAD2.1&2.2, ZmFAD7, and ZmSLD1&3) in maize were significantly up-regulated under cold stress [15]. Barrero-Gil et al. (2016) found that tomato plants improved their tolerance of low temperature by a chilling acclimation process entailing comprehensive transcriptional and metabolic adjustments, including the accumulation of compatible solutes and mobilization of antioxidant systems. However, few studies had been conducted from the transcription and metabolism levels to explore cold acclimation mechanism of the bitter gourd.

The objective of this study was to reveal the cold acclimation mechanism of bitter gourd. Cold-susceptible bitter gourd (Y17) and cold-resistant ones (Y54) were cultivated under 5 °C for 24 h and a multidisciplinary approach, including phenotypic, physiological and genetic and metabolic analysis methods, was used to reveal anti-could mechanism of cold-resistant bitter gourd. These data could provide meaningful and practical information on understanding physiological mechanisms of bitter gourd underlying this adaptive response and providing a theoretical and practical basis for breeding low-temperature tolerant cultivars.

Materials and methods

Plant materials and growth conditions

Two bitter gourd cultivars (cold-susceptible Y17) and cold-resistant Y54) were screened in our previous work and used as the materials in this study. After germination, bitter gourd seedlings were grown in pots filled with sterilized soil, and they were kept in a plant growth chamber under 12 h photoperiod with 60±5 % relative humidity, temperature 25±1 % and 150 μmol photons m^{-2} s^{-1} light intensity. When the seedlings grew to three leaves and one heart period, uniform bitter gourd seedlings (the number of each cultivar was 70) were placed in a growth chambers and subjected to low temperature treatment (5±1 °C) with light intensity of 150 μmol m^{-2} s^{-1} and 12 h

photoperiod for 24 h. The leaves of six random samples of each bitter gourd cultivar were harvested after 0, 1, 2, 4, 8, 12 and 24 h of low temperature treatment. Leaf samples were either used as fresh or were immediately frozen in liquid nitrogen and stored at -80 °C before measurements.

Determination of physiological indices at different time interval

About 0.2 g fresh leaf segments collected from each pot at different time intervals were used for the determination of proline, superoxide dismutase (SOD), (peroxidase) POD, catalase (CAT), hydrogen peroxide (H_2O_2) and malondialdehyde (MDA). The proline content was determined using a sulfosalicylic acid assay. CAT activity was determined according to method of Wani et al. (2018). Concentration of MDA was assessed by a thiobarbituric acid colorimetric method [2]. POD activity was determined by the method described by Zhang et al. (2005). SOD activity was estimated by the method of nitroblue tetrazolium assay [16]. H_2O_2 content was assayed by the method described by Si et al. (2017).

Gene expression analysis

For RNA extraction and cDNA synthesis, total RNA was extracted from fresh leaves by using trizol reagents (Invitrogen, Carlsbad, CA, USA) according to manufacturer's protocols, and DNase I (Roche) was used to purify total RNA. After extraction, RNA samples were utilized to construct the cDNA libraries, each library being generated from an equivalent mixture of three independent RNA preparations. Constructing the libraries and subsequent sequencing was performed by Illumina HiSeq TM 2000 technology supplied by NovoGene corporation (Beijing, China).

Quantification of genes: HTSeq v0.6.1 was used to count the reads numbers mapped to each gene. And then FPKM of each gene was calculated based on the length of the gene and reads count mapped to this gene. FPKM, expected number of Fragments Per Kilobase of transcript sequence per Millions base pairs sequenced, considers the effect of sequencing depth and gene length for the reads count at the same time, and is currently the most commonly used method for estimating gene expression levels [18].

Differentiation of expressed genes: Differential expression analysis of two groups was performed by using the DESeq R package (1.18.0). DESeq provide statistical

routines for determining differential expression in digital gene expression data by using a model based on the negative binomial distribution. The resulting *P*-values were adjusted using the Benjamini and Hochberg's approach for controlling the false discovery rate. Genes with an adjusted *p*-value <0.05 found by DESeq were assigned as differentially expressed.

Metabolite extraction and metabolite profiling analysis

For each bitter gourd variety, the leaves of six random samples at 0 h, 8 h and 24 h after the treatment were chosen to analyze their initial metabolites' changes. After freeze-drying, the leaf tissue was grinded to powders. Approximately 150 mg tissue powders were transferred into 2 ml centrifuge tubes. 1mL mixed solution contained methanol, acetonitrile and water (2:2:1, *v/v/v*) was added to the tubes. The mixtures were vortexed at low temperature (4℃) for 30 min, twice. Subsequently, the tubes were placed at -20 °C for 1 h. The supernatant was decanted into a new glass tube and freeze-dried after centrifuging at 13,000 rpm (4 °C) for 15 min. Then, the samples were saved under -80 °C condition. Before GC-MS analysis, 100 uL of mixed solution contained acetonitrile and water (1:1, v/v) to the samples to vortex. After centrifuging at 14,000 rpm (4 °C) for 15 min, the supernatant was extracted to analysis. The samples were placed in the 4 °C autosampler during all analysis process. Agilent 1290 Infinity LC Systems was used to separate and chromatographic column HILIC was used to separate. The injection volume was 2 uL, the flow rate was 0.3 mL/min, and the column temperature was 25 °C, respectively. The mobile phase A was water consisted of 25 Mm ammonium acetate and 25 Mm ammonia. Mobile phase B was acetonitrile.

Original data was converted into the format of mzXML by ProteoWizard. Then XCMS program was used to extract peak area. Metabolites were identified by searching the database established by our laboratory. SIMCA-P 14.1 software package (Umetrics, Umea, Sweden) was conducted a principal component analysis (PCA), partial least squares discriminant analysis (PLS-DA), orthogonal partial least squares discriminant analysis (OPLS-DA) after the data treated by Pareto-scaling. PLS-DA and OPLS-DA analyses were used to obtain variable importance values (VIP). Differential metabolites were found using Student's *t* test ($p<0.05$) and VIP (VIP>1.2).

Statistical analysis

In this study, differentiation between two groups were analyzed by utilizing the Student's t-test. Spearman's correlation was performed to analyse the correlation by SPSS 18.0. A P value less than 0.05 was consider as statistical significance.

Results

Morphology changes in response to low temperature stress

The low temperature exerted remarkably inhibition effects on the growth of bitter gourd seedlings. As shown in **Figure 1**, the damage of low temperature to the cold-susceptible bitter gourd Y17 seedlings was obvious after 2 h of 5 °C exposure. The leaves of Y17 seedlings appeared the leaves rolling and water loss. Many Y17 seedlings died when low temperature treatment was prolonged to 24 h. However, the obvious damage of low temperature on the morphology of Y54 seedlings appeared after exposing to low temperature for 8 h. After 24 h of the treatment, Y54 seedlings were still survival and but the leaves were rolled and water loss was also observed.

Changes in MDA and H_2O_2 content in response to low temperature

With increasing times exposed to the low temperature, the content of MDA and H_2O_2 in two bitter gourds were both increased (**Figure 2**). After 24 h of low temperature treatment, MDA content of the bitter gourd Y17 and Y54 were increased 97.80% and 89.29% as compare with them before the cold treatment, respectively ($P < 0.05$). After the treatment for 8 h, 12 h and 24 h, Y54 had a lower content of MDA and H_2O_2 than Y17 ($P < 0.05$). The content of MDA in Y54 was 3.65±0.51 umol g^{-1} at 24 h after the low temperature treatment, which was lower than Y17 by 19.41%. Maximum contents of H_2O_2 (2.48±0.074 mmol g^{-1}) were observed in the Y17 after 24 h cold treatment, which was also higher than Y54 ($P < 0.05$).

Changes in enzymatic activities and proline content in response to low temperature

As shown in **Figure 3,** the activities of POD, SOD and CAT and the content of proline content of in the bitter gourd seedlings were evaluated at different time points after low temperature treatment. With the increased time exposed to low temperature,

four indexes of bitter gourd Y17 and Y54 were exerted as an increasing trend, especially at 8 h after treatment. At 24 h after the cold treatment, POD, SOD and CAT activity of Y17 were 101.67±9.60 U $g^{-1}min^{-1}$, 219.48±2.52 U g^{-1} and 26.47±0.017 U g^{-1}, respectably. They were significantly higher than that before the treatment by 69.44%, 100.02% and 69.61%, respectively ($P < 0.05$).

By comparison with Y17, Y54 presented significantly higher activities of POD, SOD and CAT and the content of proline after exposing to low temperature. The POD activity of Y54 was found to be 61.67±2.45 U $g^{-1}min^{-1}$ at the beginning of exposure to low temperature, while it increased by 40.54% after 4 h, 72.97% after 8 h and 104.32% after 24 h of 5 °C exposure, respectively (**Figure 3A**). The POD activity of the bitter gourd Y54 was significantly higher than Y17 by 23.93% after 24 h low temperature treatment ($P < 0.05$). The SOD activity of Y54 at 5 °C was found to be 103.09±5.17 U g^{-1} at beginning of the treatment, but this activity was increased to 225.64±2.31 U g^{-1} after 24 h cold treatment (**Figure 3B**). As compared to the beginning treatment, CAT activity of Y54 after 24 h treatment was elevated by 81.73%, and which was also higher than Y17 by 9.25% (**Figure 3C**). After growing at 5 °C for 24 h, the proline content of Y54 was 51.16±1.58 mg g^{-1}, and it was increased by 114.32% as to compare with them at 0 h. The proline content of Y54at 24h was also higher than Y17 by 17.85% (Fig 3D).

H_2O_2 content of the bitter gourd showed a significant positive correlation with MDA content (r=0.48, $P < 0.05$), with POD activity (r=0.54, $P < 0.05$), with SOD activity (r=0.54, $P < 0.05$). But no significant correlation was found between H_2O_2 content and CAT activity (**Table 1**).

Table 1 the relationship of physiological indexes

	Proline	MDA	SOD	POD	CAT	H_2O_2
Proline	1	0	0	0	0	0
MDA	0.17	1	0	0	0	0
SOD	0.56*	0.042	1	0	0	0
POD	0.64**	0.28	0.80**	1	0	0
CAT	0.50*	-0.16	0.053	0.25	1	0
H_2O_2	0.53*	0.48*	0.54*	0.54*	-0.085	1

* Indicates a significant difference at the 0.05 level. ** Indicates a significant difference at the 0.01 level.

Differentially expressed genes in response to low temperature stress

In this study, 46 differentially expressed genes associated with cold acclimation were selected and annotated after the bitter gourd exposed at 5 °C (**Figure 4**). Among them, 69.57% (n=32) of genes encoded transcription factors, 8.70% (n=4) encoded peroxidaseand others encoded SOD, sucrose synthase, pyruvate decarboxylase, glutamate dehydrogenase, chalcone synthase, phosphofructokinase, nerolidol synthase and germacrene D synthase. As compared to 0 h of the treatment, four POD genes in the Y54 showed 5 to 25 folds than that at 8 h after low temperature treatment, and 6 to-452 folds than than at 24 h, respectively. However, three of them in the Y 17 were alos showed increased gene expression level at 8 h after the treatment, and increased levels ranged from 0.28-1.05 folds. Continued low temperature treatment for 24 h led to down-regulated expression of three POD genes. SOD gene of Y17 at 8 h and 24 h after low temperature treatment showed 1.36- and 5.52-fold increased gene expression level, respectively. In Y54, gene expression level of SOD gene increased 2.35 and 11.02 folds. After 8 h low temperature treatments, there were 27 and 23 up-regulated expression of transcription factor genes in Y54 and Y17, respectively. While the numbers of up-regulated expression genes changed to 27 and 25 after 24 h of the low temperature treatment. The elevated gene expression levels that were higher in the Y54 than that in the Y17 consisted of mainly transcription factor after exposing to low temperature for 8 h and 24 h, including McERF, McMYB, McWRKY, McTCP, McRAX, McPCL, McGATA, McE2F and McbZIP (**Figure 4**).

Differentially expressed metabolites in response to low temperature stress

Based on the metabolic responses of Y54 and Y17 under low temperature stress, 195 differential expressed metabolites, including 48 amino acids, 2 polypeptides, 35 sugars, 10 fatty acids, 38 organic acids, 11 flavonoids, 7 nucleobases, 4 coenzyme, 1 steride and 39 other compounds were screened out and analyzed. Among them, 132 metabolites had greater accumulation levels in the Y54 and Y17 after 8 h and 24 h low temperature treatment, including 37 amino acids, 2 polypeptides, 33 sugars, 3 fatty acids, 22 organic acids, 1 flavonoid, 7 nucleobases, 3 coenzyme, 1 steride and 22 other compounds. As compared to the bitter gourd Y17, 115 metabolites had greater

accumulation levels in Y54 after exposing to low temperature for 8 h and 24 h., including 35 amino acids, 24 sugars, 10 fatty acid, 19 organic acids, 1 flavonoid, 7 nucleobase, 2 coenzyme, 1 steride and 16 other compounds. A further comparative analysis indicated the main difference expression of sugar in the bitter gourd Y54 and Y17 were sucrose and raffinose (**Figure 5**).

Discussion

Damages of low temperature stress on the growth of bitter gourd seedlings

Low temperature is a major environmental stress seriously affects plant growth, distribution and productivity [1]. Many crops originated from tropical origin are highly sensitive to low non-freezing temperatures due to their bad chilling adjustment ability [3]. In this work, our results showed that low temperature had a serious damage to the growth of bitter gourd. Sustained low temperature exposing even caused the death of cold-susceptible bitter gourd seedlings (**Figure 1**). Plant cells produce excess of reactive oxygen species (ROS) under low temperature stress, and then leaded to the accumulation of MDA. MDA produced in membranes could destroy membrane structure and cause protein degradation, and then it aggravated by the damage of low temperature [19-20]. After the low temperature treatment, MDA in the bitter gourd Y17 presented an time-dependent trends. Content of MDA was increased almost 1-fold after 24 h low temperature stress access (**Figure 2**). By comparison with cold-susceptible bitter gourd Y17, the cold-resistant bitter gourd Y54 had a good ability to counter low temperature stress (**Figure 1**). After 24 h of low temperature stress, the leaves of Y54 presented a little roll and water loss. We found that this adaptive response exerted extensive transcriptional and metabolic adjustments, such as the accumulation of osmoregulation substance, the mobilization of antioxidant systems and the increasing expression of transcription factor genes.

Cold acclimation in bitter gourd results in mobilization of antioxidant mechanisms

For keeping the balance of ROS numbers, plants had evolved some enzymatic and non-enzymatic antioxidant systems to eliminate or reduce the ROS damage induced by low temperature stress [1, 21]. SOD, POD and CAT were considered as the most important antioxidant enzymes to eliminate ROS. The main function of SOD is

converting highly reactive O_2^- to O_2 and H_2O_2. POD, CAT could disintegrate produced H_2O_2 into water and molecular oxygen [21-22]. Plants usually elevated antioxidant enzyme levels to tolerance various environmental stresses [1, 6]. Similarly, the significant enhances of the activity of POD, SOD and CAT was detected in the bitter gourd Y17 and Y54 after exposure to low temperature for 24 h (**Figure 3**). The phenomenon of elevated antioxidant enzyme activities also found in other crops when they were exposed to different environmental stresses, such as maize [24] and tomato [25]. This was a normal defensive response of the plant to low temperature stress, which was benefit to the plant with higher cold tolerance to survival in the colder environment or a long time of low temperature stress [26-27]. The correlation analysis also showed that there was a significant positive correlation between H_2O_2 content and MDA content, SOD and POD activities (**Table 1**). In this study, low temperature stress caused the accumulation of MDA content leading to the elevated the SOD activity to convert ROS to H_2O_2 in bitter gourd. And then, enhanced the activity of POD and CAT eliminated the content of H_2O_2.

The antioxidant defense system involved in resisting low temperature stress in bitter gourd could also be detected at the transcript level (**Figure 3**). In this study, POD genes displayed an increased transcript expression in the bitter gourd Y54 after 8 h and 24 h after the low temperature treatment. Significantly increased POD gene expression was also reported during low temperature stress in case of bermudagrass [7]. The expression level of genes encoding SOD enzyme in the bitter gourd Y54 increased 2 to 11 folds after exposing to the low temperature. Similarly, low temperature stress was reported to up-regulate the expression of SOD in rice [28] and bermudagrass [29].

By comparison with the cold-susceptible bitter gourd Y17, the MDA and H_2O_2 content in Y54 were significantly lower after low temperature treatment (**Figure 2**). It implied that damages by cold stress in the bitter gourd Y54was weaker than Y17. The result showed that the reason for the cold-resistant bitter gourd presented better ability of adapting to low temperature stress was attributed to its higher antioxidant enzyme gene expression levels and activities in response to low temperature (**Figure 3, 4**). As compared to Y17, activities of POD, SOD and CAT in the bitter gourd Y54 were

increased by 2.80% - 23.93% after 24 h low temperature stress treatment. The expression of three POD genes was down-regulated at the time point of 24 h treatment, while all POD genes were up-regulated. Therefore, better antioxidant defense system could help the plant to preferably adapt to the condition of low temperature stress.

Cold acclimation response in bitter gourd involves transcription factors associated with cold acclimation

Transcription factor was a critical element of all gene regulatory networks that mediated various aspects of plant growth and developmental processes. It also played a central role in controlling the expression of cold-responsive genes in cold acclimation [30-31]. The results showed that the expression levels of most transcription factor genes were up-regulated in the bitter gourd subjected to 5 °C for 8 h and 24 h (**Figure 4**). These findings strongly suggested that the transcription factor played an important role in cold acclimation process in bitter gourd. These differentially expressed genes associated with cold acclimation encoded transcription factors could also find in other cold tolerant plant [3, 32]. The research of Satoh et al. (2004) found that bZIPs transcription factor had potential ability to activate the expression of ProDH gene, which participated in the metabolism of proline in Arabidopsis plants. In this study, the expression levels of McbZIP1 and McbZIP2 transcription factors of the bitter gourd Y54 were up-regulated after exposing to the low temperature (**Figure 4**). It suggested that McbZIPs transcription factor participated in the response of the bitter gourd to low temperature stress. WRKY transcription factor genes had been known as important roles in the regulation of transcriptional reprogramming associated with plant stress responses [32]. For example, transgenic Arabidopsis plants overexpressing GmWRKY21 showed increased tolerance to cold stress when compared with wild-type plants [34]. This study found that four WRKY transcription factor genes were all elevated the transcription expression level after exposing to the low temperature. Therefore, it indicated that the WRKY transcription factors exerted an important role in promoting the bitter gourd to resist low temperature stress. Some researches had demonstrated that the family of MYB transcription factor involved in the expression of ABA-dependent gene and CBF genes, and then influence the performance of the plant

to resist low temperature stress [32, 35]. Six MYB transcription factor gene up-regulated expression levels after 8 h and 24 h of the low temperature treatment, which suggested that these transcript factor had a close tine to the cold tolerance mechanism in the bitter gourd.

Cold-induced osmoregulation substance expression

In response to the low temperature stress, the plant accumulated a range of osmoregulation substance to resist the cold stress, including sugar, fatty acid and amino acid [8, 32]. A lot of evidence had demonstrated that there was a significant positive correlation between the accumulation of proline and improved cold tolerance in the plant [3, 36]. Proline can regulate cytosol acidity and decrease lipid peroxidation to improve plant tolerance of low temperature stress [37]. In this study, there was an obvious increasing trend of the proline content in the bitter gourd after exposing to the low temperature stress (**Figure 3**). It suggested that the bitter had the ability of improving the proline contents to resist the cold stress. We also found that the glutamate which participated in synthesizing the proline was significantly improved after exposing to the low temperature stress (**Figure 5**). In addition, the cold-resistant bitter gourd Y54 could accumulate more proline than the cold-susceptible bitter gourd Y17 after 2 h treatment, which suggested that the cold-resistant bitter gourd had a faster and better chilling acclimation to cold stress.

Carbohydrate metabolism had been reported to be a main contributed process of chilling acclimation, and the precursors or intermediate products of carbohydrate compounds all could play roles as osmoregulators, cryoprotectants or signaling molecules [38-39]. Soluble carbohydrate could regulate cell osmotic potential to maintain the balance of osmotic pressure, and protect plasma membrane during cold stress [40]. In this study, 33 sugars had up-regulated expression levels in the bitter gourd after 8 h and 24 h low temperature treatment. These up-regulations suggested that there was a tight link between carbohydrate metabolism and cold stress. The bitter gourd could adjust carbohydrate metabolism to tolerant cold stress. The accumulation of sucrose as an osmoprotectant supported the role of the plant to stabilize cellular membranes and maintains turgor, when they exposed to low temperature stress [41].

The metabolites profiling analysis showed that the sucrose had greater accumulation levels in the bitter gourd at 8 h and 24 h after low temperature treatment. Meanwhile, the expression level of sucrose synthase was up-regulated, which suggested that the sucrose metabolism also presented an important role in chilling acclimation of the biter gourd. After exposing to the low temperature stress for 8 h and 24 h, the expression level of raffinose in the bitter gourd has significantly elevated (**Figure 5**). This phenomenon of increased raffinose content also could be found in other plant subjected to cold stress, such as maize, Siberian spruce [42-43]. The role of the raffinose has been implicated in membrane protection and radical scavenging, and the accumulation of raffinose content is contributed to elevate cold tolerance of the plant [44-45]. Therefore, carbohydrate metabolism plays an important role in chilling acclimation of the bitter gourd.

Conclusions

In this study, the result demonstrated that the low temperature stress had obvious damages to the growth of the chilling sensitive bitter gourd. However, the bitter gourd also developed some protected mechanisms to tolerance the cold stress. The defense mechanisms included the accumulation of osmoregulation substance, such as amino acid, sugar, fatty acid and organic acid, and the mobilization of antioxidant systems, such as up-regulating expression levels of genes related cold tolerant and increasing antioxidant enzyme activities. In addition, chilling acclimation was regulated by a similar set of transcription factors, including bZIP, MYB and WRKY family. The cold-resistant bitter gourd has a better performance than the cold-susceptible bitter gourd in these aspects. Based on the result, we expect that this work can provide new knowledge and idea to breeding cold tolerance bitter gourd.

Acknowledgments

This work was financially supported by Key Research and Development Projects of Hainan Province（ZDYF2019103）and National Nonprofit Institute Research Grant of CATAS-TCGRI (1630032017027).

Author Contributions

Writing - original draft: Yu Niu, Ziji Liu, Yan Yang.

Writing – review & editing: Yan Yang, Zhiqiang Qi.

Performed the experiments: Huang He.

Analyzed the data: Xu Han

References

1. Wani W A, Jan N, Qazi H A, Andrabi K I, John R. Cold stress induces biochemical changes, fatty acid profile, antioxidant system and gene expression in Capsella bursa pastoris L.. Acta Physiologiae Plantarum. 2018; 40: 167.
2. Peng M W, Chang Y L, Chu G M, Wang M. Low-temperature tolerance and transcriptome analyses during seed germination of Anabasis aphylla. Journal of Plant Interactions. 2019; 14(1): 254-264.
3. Barrero-Gil J, Huertas R, Rambla LJ, Granell A, Salinas J. Tomato plants increase their tolerance to low temperature in a chilling acclimation process entailing comprehensive transcriptional and metabolic adjustments. Plant, Cell and Environment. 2016; 39: 2303-2318.
4. Li S M, Yang Y, Zhang Q, Liu N F, Xu Q G, Hu L X. Differential physiological and metabolic response to low temperature in two zoysiagrass genotypes native to high and low latitude. PLoS ONE. 2018; 13(6): e0198885. https://doi.org/10.1371/journal.pone.0198885
5. Caverzan A, Passaia G, Rosa S B, Ribeiro C W, Lazzarotto F, Margis-Pinheiro M. Plant responses to stresses: role of ascorbate peroxidase in the antioxidant protection. Genetics and Molecular Biology. 2012; 35(4): 1011-1019.
6. Raja V, Majeed U, Kang H, Andrabi KI, John R. Abiotic stress: interplay between ROS, hormones and MAPKs. Environmental and Experimental Botany. 2017; 137: 142-157.
7. Liu A, Hu Z G, Bi A Y, Fan J B, Gitau M M, Amombo E, et al. Photosynthesis, antioxidant system and gene expression of bermudagrass in response to low temperature and salt stress. Ecotoxicology. 2016; 25(8): 1445-1457.

8. Ruelland E, Vaultier M N, Zachowski A, Hurry V, Kader J C, Delseny M. Cold signalling and cold acclimation in plants. Advances in Botanical Research. 2009; 49: 35-150.
9. Theocharis A, Clément C, Barka E A. Physiological and molecular changes in plants grown at low temperatures. Planta. 2012; 235: 1091-1105.
10. Lucas E A, Dumancas G G, Smith B J, Clarke S L, Arjmandi B H. Chapter 35 - Health Benefits of Bitter Melon (Momordica charantia). In R. R. W. R. Preedy (Ed.), Bioactive Foods in Promoting Health. San Diego: Academic Press. 2010. pp. 525-549.
11. Saini R, Rai K P, Bara M B, Sahu P, Anjer T, Kumar R. Effect of different seed priming treatments and its duration on seedling characters of Bitter gourd (*Momordica charantia* L.). Journal of Pharmacognosy and Phytochemistry. 2017; 6(5): 848-850.
12. Wang J N, Shang S, Tian L B, Zhou M M, Pan Q Y, Zou K X, Du Z T. Effects of low temperature stress on antioxidant system of grafted bitter gourd seedlings. Chinese Journal of Tropical Crops. 2018; 39(2): 237-245. (With Chinese abstract)
13. Niu Y, Liu Z J, Liu Z H, Li H F, Fu J J, Yang Y. Identification of cold tolerance species of bitter gourd. Northern Horticulture.2017;24:1-6.
14. Zhao J L, Zhang S H, Yang T F, Zeng Z C, Huang Z H, Liu Q, et al. Global transcriptional profiling of a cold-tolerant rice variety under moderate cold stress reveals different cold stress response mechanisms. Physiologia Plantarum. 2015; 154(3): 381-394.14. Zhao X C, Wei J P, He L, Zhang Y F, Zhao Y, Xu X X, et al. Identification of fatty acid desaturases in maize and their differential responses to low and high temperature. Genes. 2019; 10: 445.
15. Zhang H, Jiang Y, He Z, Ma M. Cadmium accumulation and oxidative burst in garlic (Allium sativum). Journal of Plant Physiology. 2005; 162: 977-984.
16. Si T, Wang X, Wu L, Zhao C Z, Zhang L N, Huang M, et al. Nitric Oxide and Hydrogen Peroxide Mediate Wounding-Induced Freezing Tolerance through Modifications in Photosystem and Antioxidant System in Wheat. Frontiers in Plant Science. 2017; 8: 1284.

17. Trapnell C, Williams B A, Pertea G, Mortazavi A, Kwan G, van Baren M J, et al. Transcript assembly and quantification by RNA-Seq reveals unannotated transcripts and isoform switching during cell differentiation. Nature Biotechnology. 2010; 28: 511-515.
18. Langridge P, Paltridge N, Fincher G. Functional genomics of abiotic stress tolerance in cereals. Briefings in Functional Genomics & Proteomics. 2006; 4: 343-354.
19. Martinez V, Nieves-Cordones M, Lopez-Delacalle M, Rodenas R, Mestre T C, Garcia-Sanchez F, et al. Tolerance to stress combination in tomato plants: new insights in the protective role of melatonin. Molecules. 2018; 23(3): 535.
20. Petrov V, Hille J, Mueller-Roeber B, Gechev T S. ROS-mediated abiotic stress-induced programmed cell death in plants. Frontiers in Plant Science. 2015; 6: 69.
21. Wang F, Liu J, Zhou L, Pan G, Li Z, Cheng F. Senescence-specific change in ROS scavenging enzyme activities and regulation of various SOD isozymes to ROS levels in psf mutant rice leaves. Plant Physiology and Biochemisrty. 2016; 109: 248-261.
22. Jan N, Majeed U, Andrabi K I, John R. Cold stress modulates osmolytes and antioxidant system in Calendula officinalis. Acta Physiologiae Plantarum. 2018, 40: 73.
23. Zhu X C, Song F B, Xu H W. Influence of arbuscular mycorrhiza on lipid peroxidation and antioxidant enzyme activity of maize plants under temperature stress. Mycorrhiza. 2010; 20: 325-332.
24. Ogweno J O, Song X S, Hu W H, Shi K, Zhou Y H, Yu J Q. Detached leaves of tomato differ in their photosynthetic physiological response to moderate high and low temperature stress. Scientia Horticulturae. 2009; 123(1): 17-22.
25. Baek K H, Skinner D Z. Alteration of antioxidant enzyme gene expression during cold acclimation of near-isogenic wheat lines. Plant Science. 2003; 165(6): 1221-1227.
26. Kuk Y I, Shin J S, Burgos N R, Hwang T E, Han O, Cho B H, et al. Antioxidative enzymes offer protection from chilling damage in rice plants. Crop Science. 2003; 43(6): 2109-2117.

27. Nanculao G D, Herrera M L, Carcamo M P, Velasquez V B. Relative expression of genes related with cold tolerance in temperate rice at the seedling stage. African Journal of Biotechnology. 2014; 13(25): 2506-2512.

28. Fan J, Ren J, Zhu W, Amombo E, Fu J, Chen L. Antioxidant responses and gene expression in bermudagrass under cold stress. American Society for Horticultural Science. 2014; 139(6): 699-705.

29. Van Buskirk H A, Thomashow M F. Arabidopsis transcription factors regulating cold acclimation. Physiologia Plantarum. 2006; 126: 72-80.

30. Seo P J, Park M J, Park C M. Alternative splicing of transcription factors in plant responses to low temperature stress: mechanisms and functions. Planta. 2013; 237: 1415-1424.

31. Chen L G, Song Y, Li S J, Zhang L P, Zou C S, Yu D Q. The role of WRKY transcription factors in plant abiotic stresses. Biochimica et Biophysica Acta. 2012; 1819(2): 120-128.

32. Satoh R, Fujita Y, Nakashima K, Shinozaki K, Yamaguchi-Shinozaki K. A novel subgroup of bZIP proteins functions as transcriptional activators in hypoosmolarity-responsive expression of the *ProDH* gene in *Arabidopsis*. Plant & Cell Physiology. 2004; 45: 309-317.

33. Zhou Q Y, Tian A G, Zou H F, Xie Z M, Lei G, Huang J, et al. Soybean WRKY-type transcription factor genes, *GmWRKY13*, *GmWRKY21*, and *GmWRKY54*, confer differential tolerance to abiotic stresses in transgenic Arabidopsis plants. Plant Biotechnology Journal. 2008; 6: 486-503.

34. Agarwal M, Hao Y J, Kapoor A, Dong C H, Fujii H, Zheng X W, et al. A R2R3 type MYB transcription factor is involved in the cold regulation of CBF genes and in acquired freezing tolerance. The Journal of Biological Chemistry. 2006; 281: 37636-37645.

35. Szabados L, Savouré A. Proline: a multifunctional amino acid. Trends in Plant Science. 2010; 15: 89-97.

36. Kavi Kishor P B, Sangam S, Amrutha R N, Sri Laxmi P, Naidu K R, Rao K R S S, et al. Regulation of proline biosynthesis, degradation, uptake and transport in

higher plants: Its implications in plant growth and abiotic stress tolerance. Current Science. 2005; 88: 424-438.

37. Welling A, Palva E T. Molecular control of cold acclimation in trees. Physiologia Plantarum. 2006, 127: 167-181.

38. Fernandez O, Theocharis A, Bordiec S, Feil R, Jacquens L, Clément C, Fontaine F, Ait Barka E. Burkholderia phytofirmans strain PsJN acclimates grapevine to cold by modulating carbohydrates metabolism. International Society of Molecular Plant-Microbe Interactions. 2012; 25(4): 496-504.

39. Bogdanović J, Mojović M, Milosavić N, Mitrović A, Vučinić Ž, Spasojević I. Role of fructose in the adaptation of plants to cold-induced oxidative stress. European Biophysics Jouranl. 2008; 37(7): 1241-1246.

40. Mundree S.G., Baker B., Mowla S., Peters S., Marais S., Wilingen C.V., et al. Physiological and molecular insights into drought tolerance. African Journal of Biotechnology. 2002; 1(2): 28-38.

41. Angelcheva L, Mishra Y, Antti H, Kjellsen T D, Funk C, Strimbeck R G, et al. Metabolomic analysis of extreme freezing tolerance in Siberian spruce (Picea obovata). New Phytologist. 2014; 204(3): 545-555.

42. Shahryar N, Maaliamiri R. Metabolic acclimation of tetraploid and hexaploid wheats by cold stress-induced carbohydrate accumulation. Journal of Plant Physiology. 2016; 204: 44-53.

43. Hincha D K. Effects of calcium-induced aggregation on the physical stability of liposomes containing plant glycolipids. Biochimica et Biophysica Acta - Biomembranes. 2003; 1611(1-2): 180-186.

44. Nishizawa A, Yabuta Y, Shigeoka S. Galactinol and raffinose constitute a novel function to protect plants from oxidative damage. Plant Physiology. 2008; 147: 1251-1263.

Figures

Figure 1 Performance at the seedling stage of cold-susceptible bitter gourd Y17 and cold-resistant bitter gourd Y54 after low temperature treatment

Figure 2 Effect of cold stress on MDA and H_2O_2 content in bitter gourd seedlings at different time points. The data represent means ± SE. Bars with different letters are significantly different at $P < 0.05$ in the same time point of the cold treatment.

Figure 3 Effect of cold stress on POD, SOD and CAT activities and proline content in bitter gourd seedlings at different time points. The data represent means ± SE. Bars with different letters are significantly different at $P < 0.05$ in the same time point of the cold treatment.

Figure 4 Effects of low temperature on the cold induced gene expressions in leaves of bitter gourd

Figure 5 Differentially expressed metabolites of bitter gourd in response to low temperature stress

 Click here to access/download;Figure;Figure.docx

Figure 1

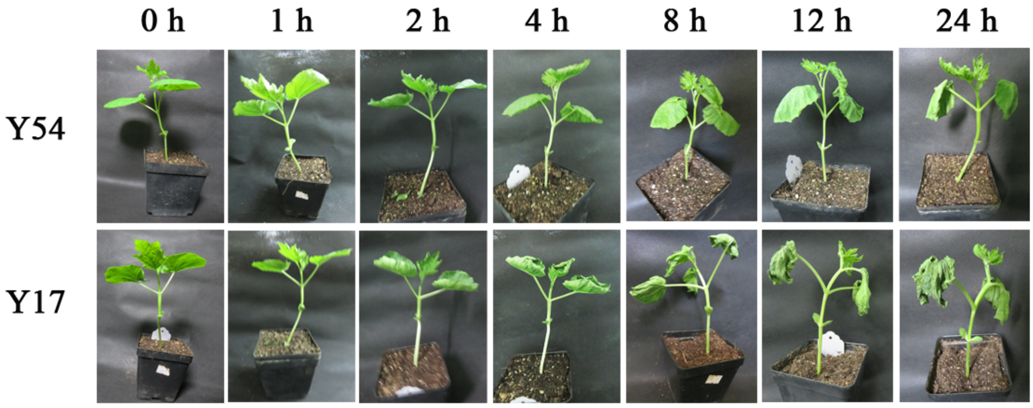

Figure 2A

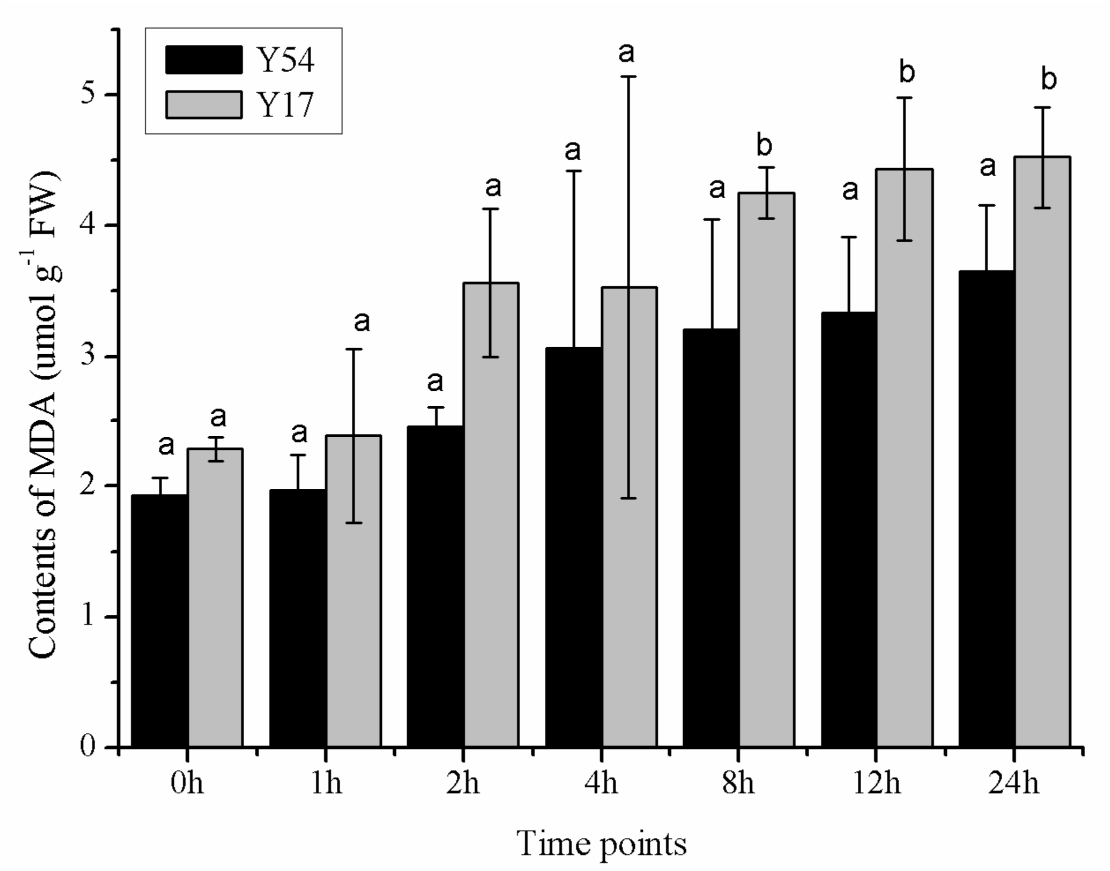

Y54
Y17
Contents of MDA (umol g-1 FW)
0
1
2
3
4
5
a
a
a
a
a
a
a
a
a
a
b
a
b
a
b
0h
1h
2h
4h
8h
12h
24h
Time points

Figure 2B

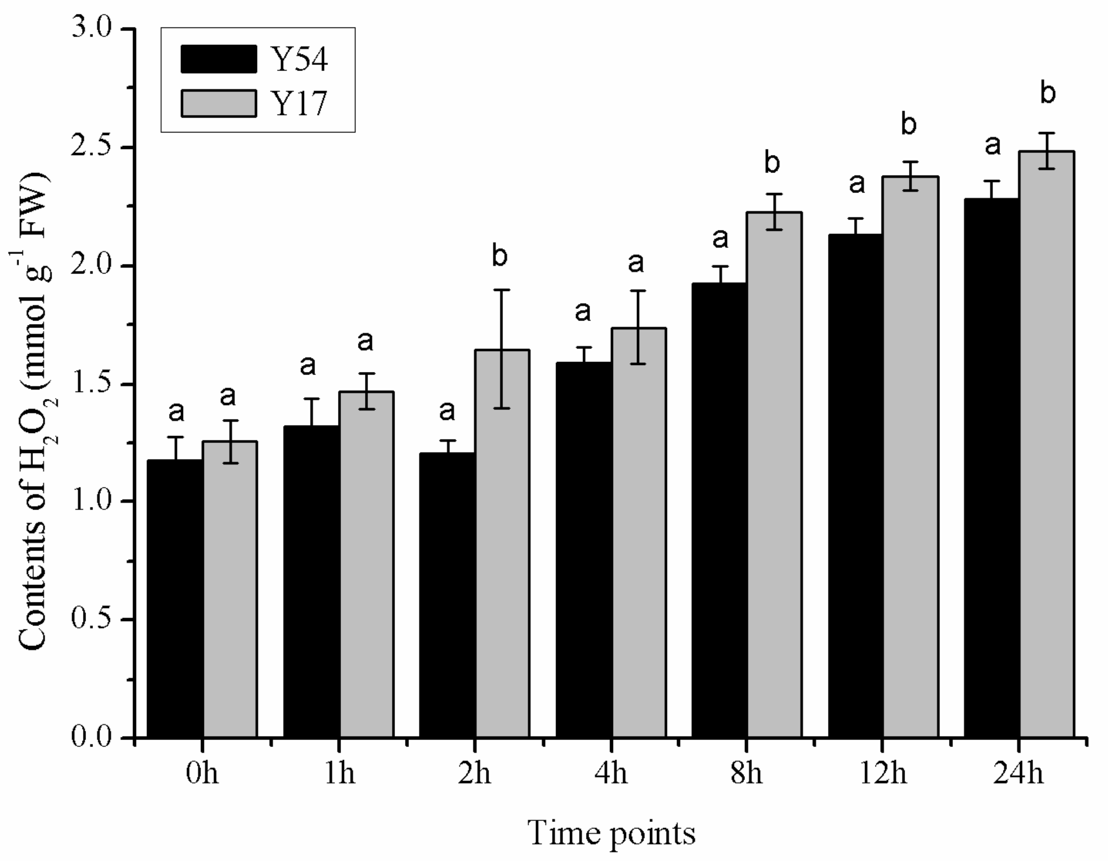

Y54
Y17
Contents of H_2O_2 (mmol g^{-1} FW)
3.0
2.5
2.0
1.5
1.0
0.5
0.0
a
a
a
a
a
b
a
a
a
b
a
b
a
b
0h
1h
2h
4h
8h
12h
24h
Time points

Figure 3A

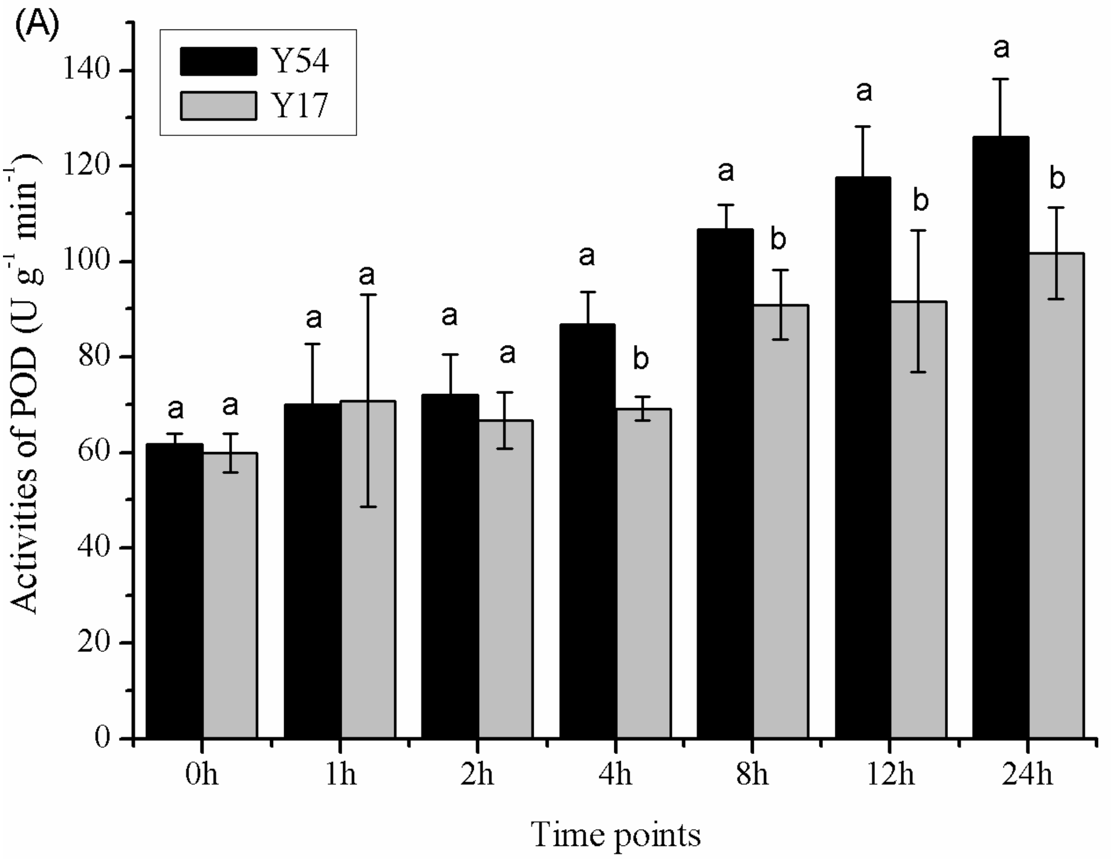
(A)
Y54
Y17
Activities of POD (U g-1 min-1)
0
20
40
60
80
100
120
140
0h
1h
2h
4h
8h
12h
24h
Time points
a
b

Figure 3B

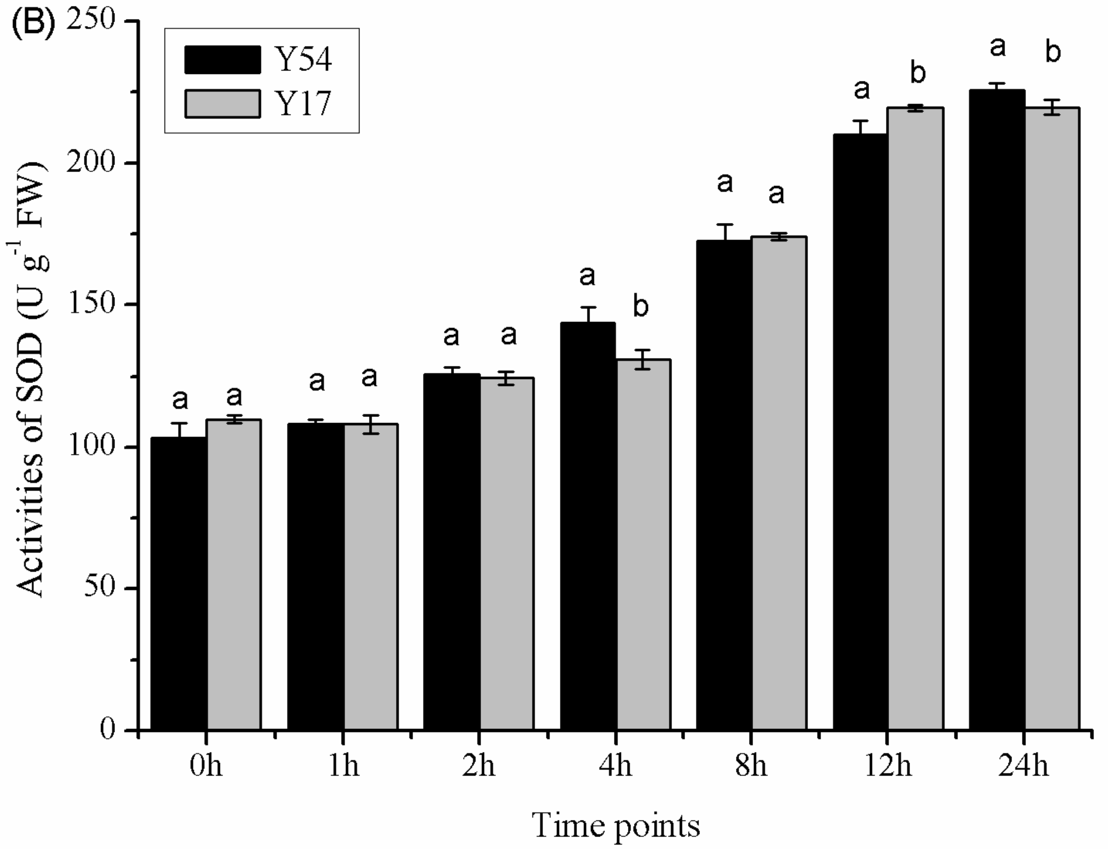

(B)
Y54
Y17
Activities of SOD (U g-1 FW)
0
50
100
150
200
250
a
a
a
a
a
a
a
b
a
a
a
b
a
b
0h
1h
2h
4h
8h
12h
24h
Time points

Figure 3C

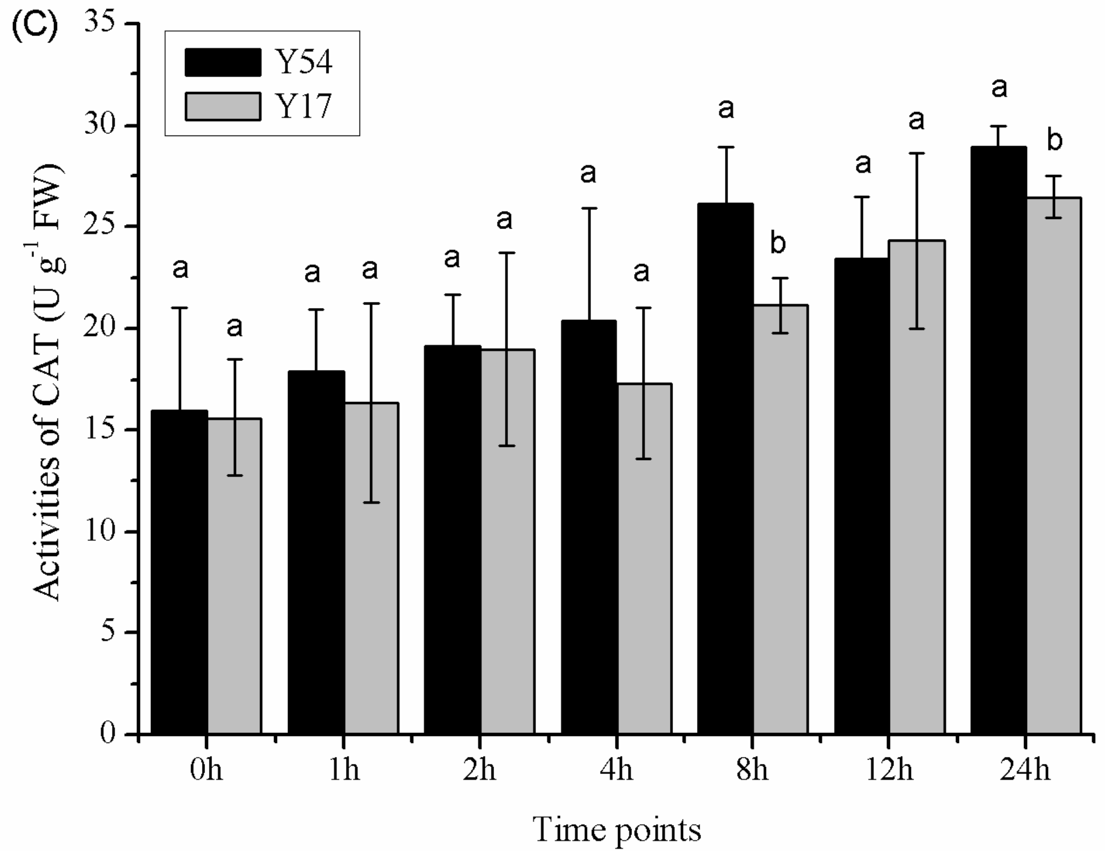

(C)
Y54
Y17
Activities of CAT (U g^{-1} FW)
Time points
0h
1h
2h
4h
8h
12h
24h
0
5
10
15
20
25
30
35
a
b

Figure 3D

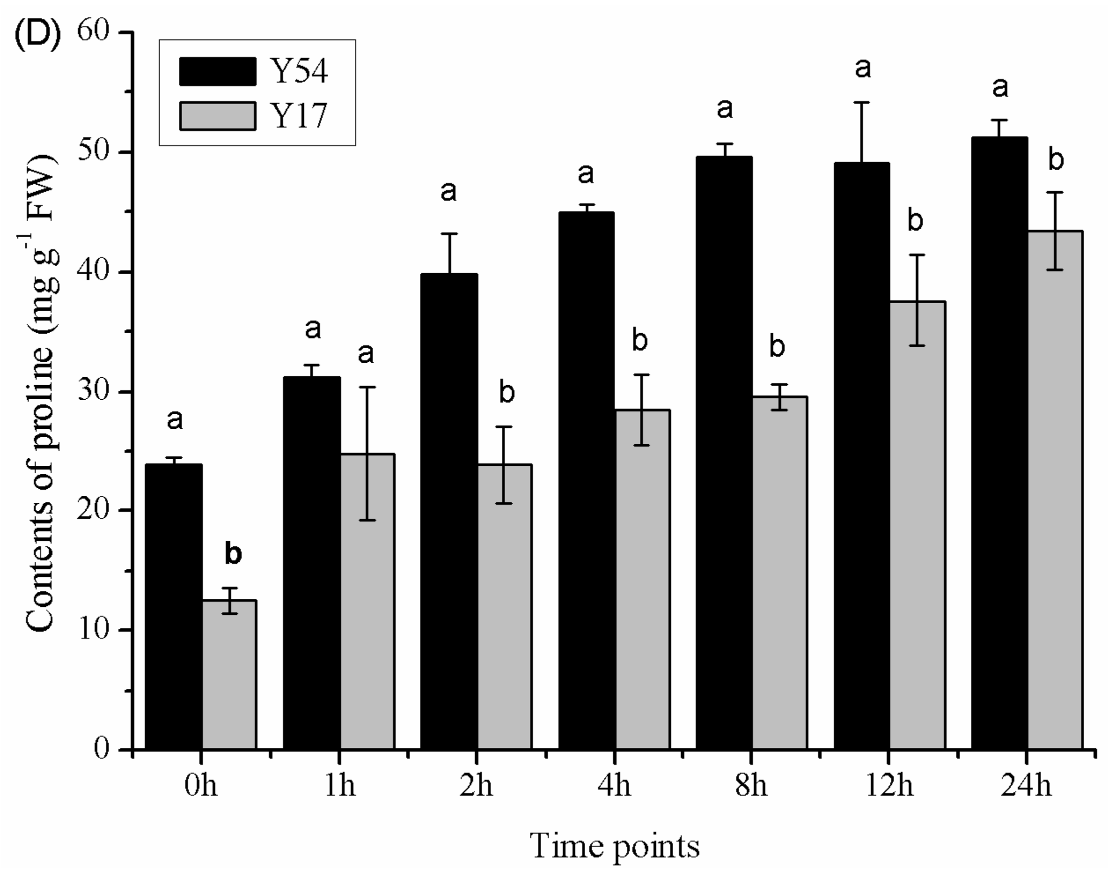

(D)
Y54
Y17
Contents of proline (mg g^{-1} FW)
0
10
20
30
40
50
60
0h
1h
2h
4h
8h
12h
24h
Time points
a
b
a
a
a
b
a
b
a
b
a
b
a
b

Figure 4

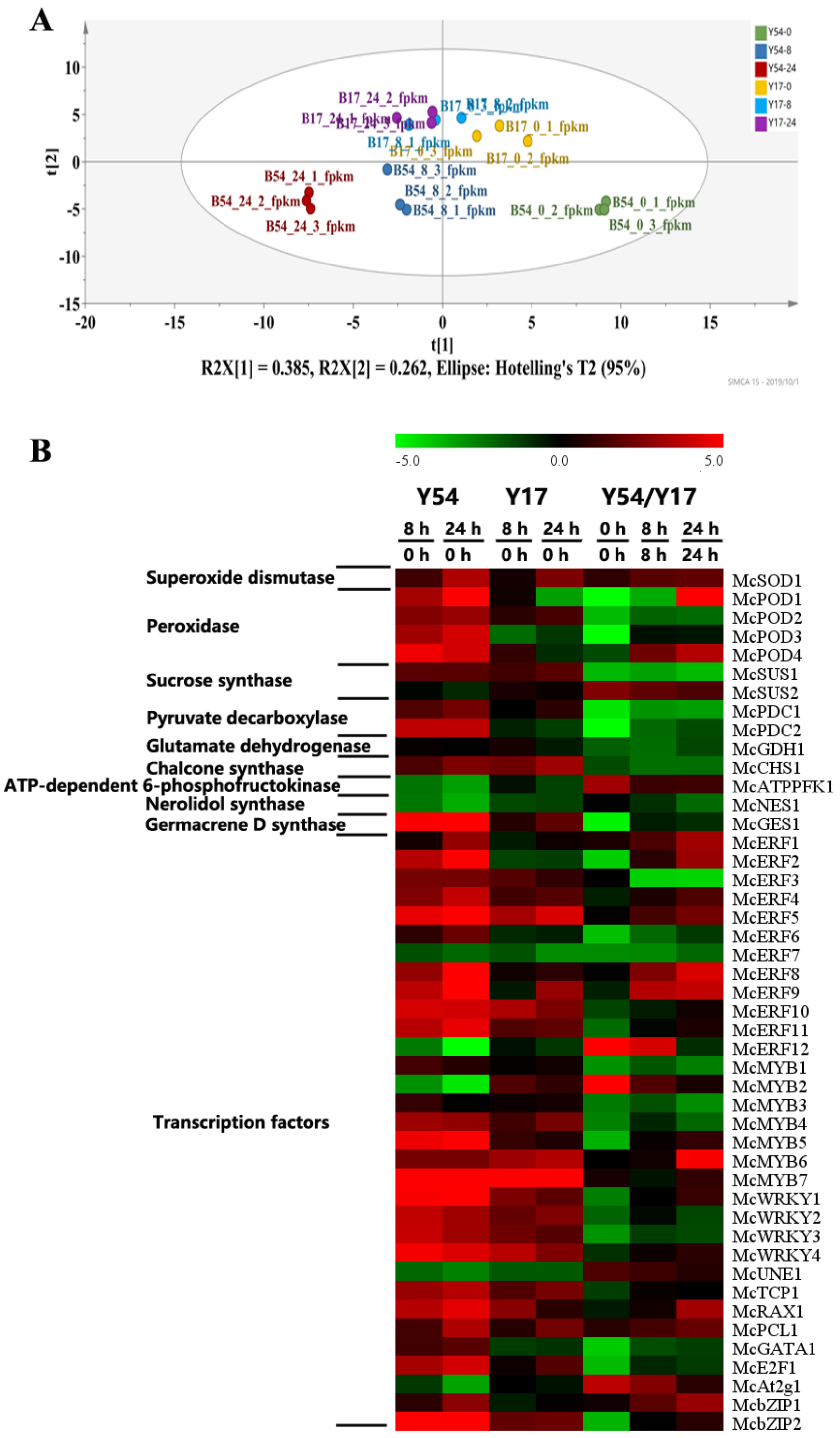

A
B17_24_2_fpkm
B17_8_1_fpkm
B17_0_1_fpkm
B17_0_3_fpkm
B17_0_2_fpkm
B54_24_1_fpkm
B54_24_2_fpkm
B54_24_3_fpkm
B54_8_3_fpkm
B54_8_2_fpkm
B54_8_1_fpkm
B54_0_2_fpkm
B54_0_1_fpkm
B54_0_3_fpkm
Y54-0
Y54-8
Y54-24
Y17-0
Y17-8
Y17-24
t[2]
t[1]
R2X[1] = 0.385, R2X[2] = 0.262, Ellipse: Hotelling's T2 (95%)
SIMCA 15 - 2019/10/1
B
-5.0
0.0
5.0
Y54
Y17
Y54/Y17
8 h / 0 h
24 h / 0 h
0 h / 0 h
8 h / 8 h
24 h / 24 h
Superoxide dismutase
Peroxidase
Sucrose synthase
Pyruvate decarboxylase
Glutamate dehydrogenase
Chalcone synthase
ATP-dependent 6-phosphofructokinase
Nerolidol synthase
Germacrene D synthase
Transcription factors
McSOD1
McPOD1
McPOD2
McPOD3
McPOD4
McSUS1
McSUS2
McPDC1
McPDC2
McGDH1
McCHS1
McATPPFK1
McNES1
McGES1
McERF1
McERF2
McERF3
McERF4
McERF5
McERF6
McERF7
McERF8
McERF9
McERF10
McERF11
McERF12
McMYB1
McMYB2
McMYB3
McMYB4
McMYB5
McMYB6
McMYB7
McWRKY1
McWRKY2
McWRKY3
McWRKY4
McUNE1
McTCP1
McRAX1
McPCL1
McGATA1
McE2F1
McAt2g1
McbZIP1
McbZIP2

Figure 5

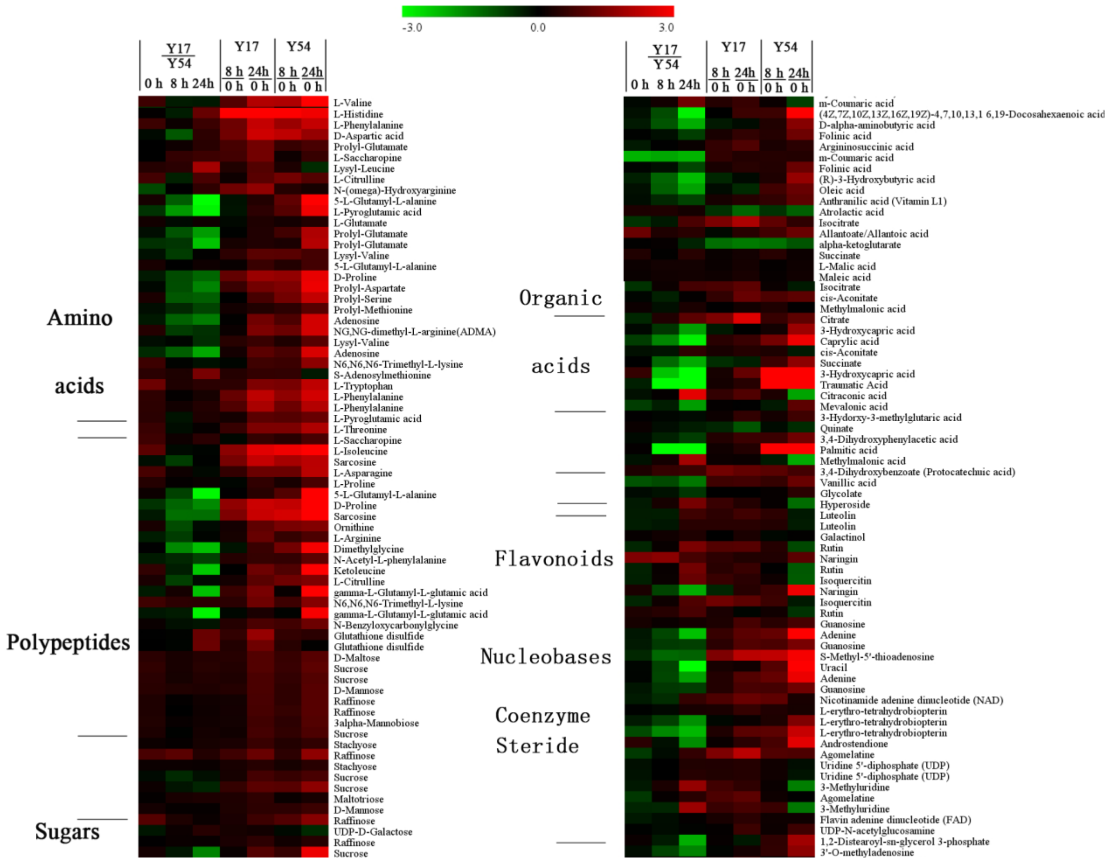
-3.0
0.0
3.0
Y17/Y54
0 h 8 h 24h
Y17
8 h/0 h 24h/0 h
Y54
8 h/0 h 24h/0 h
Amino acids
Polypeptides
Sugars
L-Valine
L-Histidine
L-Phenylalanine
D-Aspartic acid
Prolyl-Glutamate
L-Saccharopine
Lysyl-Leucine
L-Citrulline
N-(omega)-Hydroxyarginine
5-L-Glutamyl-L-alanine
L-Pyroglutamic acid
L-Glutamate
Prolyl-Glutamate
Prolyl-Glutamate
Lysyl-Valine
5-L-Glutamyl-L-alanine
D-Proline
Prolyl-Aspartate
Prolyl-Serine
Prolyl-Methionine
Adenosine
NG,NG-dimethyl-L-arginine(ADMA)
Lysyl-Valine
Adenosine
N6,N6,N6-Trimethyl-L-lysine
S-Adenosylmethionine
L-Tryptophan
L-Phenylalanine
L-Phenylalanine
L-Pyroglutamic acid
L-Threonine
L-Saccharopine
L-Isoleucine
Sarcosine
L-Asparagine
L-Proline
5-L-Glutamyl-L-alanine
D-Proline
Sarcosine
Ornithine
L-Arginine
Dimethylglycine
N-Acetyl-L-phenylalanine
Ketoleucine
L-Citrulline
gamma-L-Glutamyl-L-glutamic acid
N6,N6,N6-Trimethyl-L-lysine
gamma-L-Glutamyl-L-glutamic acid
N-Benzyloxycarbonylglycine
Glutathione disulfide
Glutathione disulfide
D-Maltose
Sucrose
Sucrose
D-Mannose
Raffinose
Raffinose
3alpha-Mannobiose
Sucrose
Stachyose
Raffinose
Stachyose
Sucrose
Sucrose
Maltotriose
D-Mannose
Raffinose
UDP-D-Galactose
Raffinose
Sucrose
Organic acids
Flavonoids
Nucleobases
Coenzyme
Steride
m-Coumaric acid
(4Z,7Z,10Z,13Z,16Z,19Z)-4,7,10,13,1 6,19-Docosahexaenoic acid
D-alpha-aminobutyric acid
Folinic acid
Argininosuccinic acid
m-Coumaric acid
Folinic acid
(R)-3-Hydroxybutyric acid
Oleic acid
Anthranilic acid (Vitamin L1)
Atrolactic acid
Isocitrate
Allantoate/Allantoic acid
alpha-ketoglutarate
Succinate
L-Malic acid
Maleic acid
Isocitrate
cis-Aconitate
Methylmalonic acid
Citrate
3-Hydroxycapric acid
Caprylic acid
cis-Aconitate
Succinate
3-Hydroxycapric acid
Traumatic Acid
Citraconic acid
Mevalonic acid
3-Hydorxy-3-methylglutaric acid
Quinate
3,4-Dihydroxyphenylacetic acid
Palmitic acid
Methylmalonic acid
3,4-Dihydroxybenzoate (Protocatechuic acid)
Vanillic acid
Glycolate
Hyperoside
Luteolin
Luteolin
Galactinol
Rutin
Naringin
Rutin
Isoquercitin
Naringin
Isoquercitin
Rutin
Guanosine
Adenine
Guanosine
S-Methyl-5′-thioadenosine
Uracil
Adenine
Guanosine
Nicotinamide adenine dinucleotide (NAD)
L-erythro-tetrahydrobiopterin
L-erythro-tetrahydrobiopterin
L-erythro-tetrahydrobiopterin
Androstendione
Agomelatine
Uridine 5′-diphosphate (UDP)
Uridine 5′-diphosphate (UDP)
3-Methyluridine
Agomelatine
3-Methyluridine
Flavin adenine dinucleotide (FAD)
UDP-N-acetylglucosamine
1,2-Distearoyl-sn-glycerol 3-phosphate
3′-O-methyladenosine

Click here to access/download
Supporting Information
Surpporting informations.docx
