# Supplementary material for: Gene expression and metabolic changes of Momordica charantia L. seedlings in response to low temperature stress
Source: PLoS One. 2020 May 29;15(5):e0233130. doi: 10.1371/journal.pone.0233130 (PMC7259686; doi:10.1371/journal.pone.0233130)
Supplement: Attachment — Submitted filename: Reviewer Attachments.docx [file pone.0233130.s001.docx]

In this study, authors investigated the cold stress response in bitter gourd at phenotypic,
physiological and metabolic levels. These results suggested that the bitter gourd exhibited higher cold tolerance may attribute to the mobilization of antioxidant systems, the adjustment of the transcription factors and accumulation of osmoregulation substance. Compared with the cold-susceptible bitter gourd, the cold-resistant bitter gourd has a better performance in these aspects.This research might provide useful information leading to how bitter gourd can adapt to the low temperature stress. However, the manuscript need to be modified based on the comments below for the publication in this journal.

1. Please increase the line number

2.Introduction：The references about cold injury are too general, please add some references from bitter gourd cultivars, herbaceous plants or vegetables, The adaptation mechanism of woody and herbaceous plants to cold injury is quite different.

3. Materials and methods:What is the basis for choosing 5 ℃ low temperature treatment?

Increase more information about materials cold-susceptible (Y17) and cold-resistant (Y54)), especially for temperature tolerance.

4.The authors assayed for change in malondialdehyde (MDA), hydrogen peroxide (H2O2), proline content and activities of antioxidant enzymes. this work provided a large amount of data, which might be informative for some potential readers.Majority of guaiacol peroxidase activity is derived from extracellular peroxidases, which are probably involved in many biological processes (such as lignin and suberin formation, cross-linking of cell wall components, and/or synthesis of phytoalexins), and non-specific peroxidases located in the vacuole. Their contribution to antioxidative defense is largely unclear. Ascorbate peroxidases and thiol-dependent peroxidases (such as peroxiredoxin), which are the representative antioxidative enzymes in plants, are much better for your study. Additional experiments, such as checking O_2_^-^ and/or ROS levels, should be added.

5. In this paper, the author sequenced the transcriptome of the two groups of samples, but there was no verification of the difference gene. Additional experiments, such as validation by qRT-PCR analysis, should be added.
