# Supplementary material for: Gene expression and metabolic changes of Momordica charantia L. seedlings in response to low temperature stress
Source: PLoS One. 2020 May 29;15(5):e0233130. doi: 10.1371/journal.pone.0233130 (PMC7259686; doi:10.1371/journal.pone.0233130)
Supplement: Attachment — Submitted filename: PONE-D-19-35964-review-20200220.docx [file pone.0233130.s002.docx]

**PLOS ONE: PONE-D-19-35964**

2020/02/21

1. Two manuscripts are received, an MS entitled by “Gene expression and metabolic changes of *Momordica Charantia* L. seedlings in response to low temperature stress”, and a New MS with the same title. There are no previous reviewer’s comments attached to the MS/New MS.
2. In general, compared with the MS and the New MS, there is not enough difference to judge which is good enough for publication with respect to the language and the results. The MS/New MS records some data of the low temperature stress experiment, but shows very limited meaningful information of the data and the results. The presentation of the results and the discussion are not complete and are not clear to me, also it is limited by its poor English language. The main problem of the work to me is the material. All the data were obtained only from the leaves instead of whole seedlings, i.e. leaves, stems, hypocotyls and roots. No arguments are found in the MS/New MS to show that the leaf material is representative of the whole seedling in the present low temperature conditions. And it is not shown whether other organs and tissues of the seedling play any roles in the stress conditions beside the leaf. Again, no data are found about the field performance and/or the condition of the plants after 24h treatment. These missing data are important to evaluate the final result of the stress, to evaluate the function of the organs, and, to evaluate the design and the timing of the experiment and sampling.
3. Some problems in the MS/New MS are as following:
   1) In the title and in the text, the Latin name of the species (*charantia* ) should not be capitalized.
   2) According to the morphology to me, Fig. 1, Y17 showed low temperature stress at 2h, equivalent to Y54 at about 12h. The MS/New MS presented the compound content or gene expression values and percentages, but showed less relation between the data and the morphology. Results should be presented with systematic comparisons in the texts in the parts of “Changes in MDA and H2O2 content in response to low temperature”, “Changes in enzymatic activities and proline content in response to low temperature”, and “Differentially expressed genes in response to low temperature stress”, or better, with figures, instead of tedious writing of percentages and folds.
   3) In Table 1, the genotype and time information should be included, and it is needed to provide arguments that the correlations are representative in the whole time length.
   4) The MS/New MS analyzed differentially expressed genes in response to the low temperature stress, and 46 differentially expressed genes associated with cold acclimation were selected. It should make clear how these genes are selected, and compared with other genes do they express in a significant level?
   5) About the Fig. 4 and Fig. 5, make clear the meaning of the titles of the figure columns such as Y17/Y54, and why some compounds are repeatedly listed, such as sucrose and rafffinose in Fig. 5.
   6) Some miscellaneous questions are labeled in the text.

Accept with Major Revision.
